# Global heliospheric termination shock strength in the solar–interstellar interaction

**DOI:** 10.1038/s41550-025-02634-3

**Published:** 2025-08-19

**Authors:** E. J. Zirnstein, R. Kumar, B. L. Shrestha, P. Swaczyna, M. A. Dayeh, J. Heerikhuisen, J. R. Szalay

**Affiliations:** 1 Department of Astrophysical Sciences, Princeton University, Princeton, NJ USA; 2 Princeton Plasma Physics Laboratory, Princeton, NJ USA; 3 Space Research Centre PAS (CBK PAN), Warsaw, Poland; 4 Southwest Research Institute, San Antonio, TX USA; 5 Department of Physics and Astronomy, University of Texas at San Antonio, San Antonio, TX USA; 6 Department of Mathematics and Statistics, University of Waikato, Hamilton, New Zealand

**Keywords:** Space physics, Astrophysical plasmas, Theoretical particle physics

## Abstract

A heliospheric termination shock (HTS) surrounds our Solar System at approximately 100 astronomical units from the Sun, where the expanding solar wind (SW) is compressed and heated before encountering the interstellar medium. HTS-accelerated particles govern the pressure balance with the interstellar medium, but little is known about the global properties of the HTS beyond in situ measurements from Voyager in only two directions of the sky. Here we fill this gap by extracting the HTS strength using particle-in-cell, test particle and magnetohydrodynamic simulations, constrained by Interstellar Boundary Explorer observations of energetic neutral atoms produced from HTS-accelerated particles. Our results reveal there is a higher compression near the poles during solar minimum compared with solar maximum due to the higher Mach number flow. North–south asymmetries arise from the disparate evolution of the polar coronal holes, while minimum compression near the flanks is probably due to SW slowing from mass loading over a greater distance to the HTS. The results imply a strong connection between the HTS strength and the SW and interstellar medium dynamics.

## Abstract (web summary)

The study reveals maps of the heliosphere’s termination shock strength. Asymmetries in the shock strength across the sky, from nose to tail and port to starboard flanks, are caused by the solar wind and interstellar medium dynamics.

## Main

The interaction of the solar wind (SW) with the local interstellar medium (LISM) forms the heliosphere, a large structure that protects us from galactic cosmic rays^1,2^ (although there is still a debate within the heliophysics community on the shape of the heliosphere, particularly the heliotail; see Kleimann et al.^3^ and references therein). Beyond the critical Alfvén point, located at around 20 solar radii^4^, the SW transitions from sub-Alfvénic to super-Alfvénic and the SW expands radially outwards from the Sun at supersonic speeds. At two to three times the distance to Pluto, a shock (that is, the heliospheric termination shock, HTS) forms before the SW encounters the partially ionized interstellar medium. The outer boundary of the heliosphere is a three-dimensional surface called the heliopause, where the interstellar plasma outside the heliopause is slowed, compressed and diverted around the heliosphere. Between the HTS and heliopause is the heliosheath (HS), containing a relatively hot plasma whose mean energy is primarily determined by the heating and acceleration of interstellar pickup ions (PUIs) at the HTS^5,6^, in the HS^7–9^ or most likely a combination of both. The location of the HTS^10–12^ and heliopause^13,14^, the plasma pressure in the HS^15–18^ and how PUIs are accelerated at the HTS are all connected, forming a quasi-pressure balance between the heliosphere and the very local interstellar medium (VLISM) that dynamically changes over time^19^.

One of the key processes in this heliospheric pressure balance is the energization of interstellar PUIs at the HTS. There have been many theoretical and modelling studies on this topic, utilizing global magnetohydrodynamic (MHD), particle-in-cell (PIC) and test particle simulations^5,6,20–22^. The acceleration of PUIs depends on the strength and microstructure of the HTS, and fortunately the Voyager 2 spacecraft provided in situ observations of the HTS structure at scales smaller than the upstream advective PUI gyroradius^23,24^. Its measurements revealed a highly dynamic shock structure with PUI foot, ramp, overshoot and undershoot. Voyager 2 crossed the HTS five times in 2007, implying a dynamically evolving shock with fast movements towards and away from the Sun. Each crossing showed slightly different kinetic-scale structures, but the compression ratios during crossings TS-2 and TS-3 are 2.38 ± 0.14 and 1.58 ± 0.71, respectively^24^, or an average of 1.98 ± 0.36. We have also learned more about PUI-mediated, quasi-perpendicular shocks from New Horizons’ Solar Wind Around Pluto (SWAP) measurements of interstellar PUIs directly, revealing that the jump conditions at interplanetary shocks cannot be accurately determined from SW ions^25,26^; rather, PUIs are the only reliable source to derive the shock conditions (besides observing the magnetic field jump, which New Horizons is not equipped to measure). One of the strongest interplanetary shocks observed by SWAP occurred in October 2015, with a compression ratio between 2.5 and 3, with characteristics qualitatively similar to Voyager 2’s observations of the HTS. Voyager 2’s Plasma Science Experiment^27^, which observed core SW particles at energies lower than those expected for PUIs^24^ (even though Plasma Science Experiment can observe particles up to 6 keV), observed lower compression ratios during TS-2 and TS-3 (~2), consistent between the SW density, speed and magnetic field jumps.

Due to their preferential acceleration at the HTS, PUIs contain a substantial fraction of the internal plasma pressure in the HS^16,17^. Energetic PUIs in the HS charge exchange with interstellar neutral atoms flowing into the heliosphere, creating energetic neutral atoms (ENAs) that propagate ballistically in all directions. Some ENAs make it to 1 au and are measured by the Interstellar Boundary Explorer (IBEX)^28^. The IBEX-Hi instrument measures ENAs in the 0.5–6 keV energy range, which approximately corresponds to the energies at which PUIs are believed to undergo ‘shock drift’ acceleration at the HTS^6^. ENA spectra observed by IBEX-Hi therefore inform us of the post-accelerated PUI distribution in the HS, and using information from models of particle acceleration at the HTS and plasma flows in the HS, we may infer the HTS compression ratio across the sky. This will help answer one of the main scientific questions of the IBEX mission: ‘What is the global strength of the HTS?’^28^, which is highly relevant to both heliophysics and astrophysics, particularly in the study of stellar–interstellar systems with supersonic stellar winds^29,30^. As with our Solar System, the shock strength will depend on the host star’s stellar wind as well as the interplay between the interstellar dynamic and magnetic pressures.

## Results

**Fig. 1:**
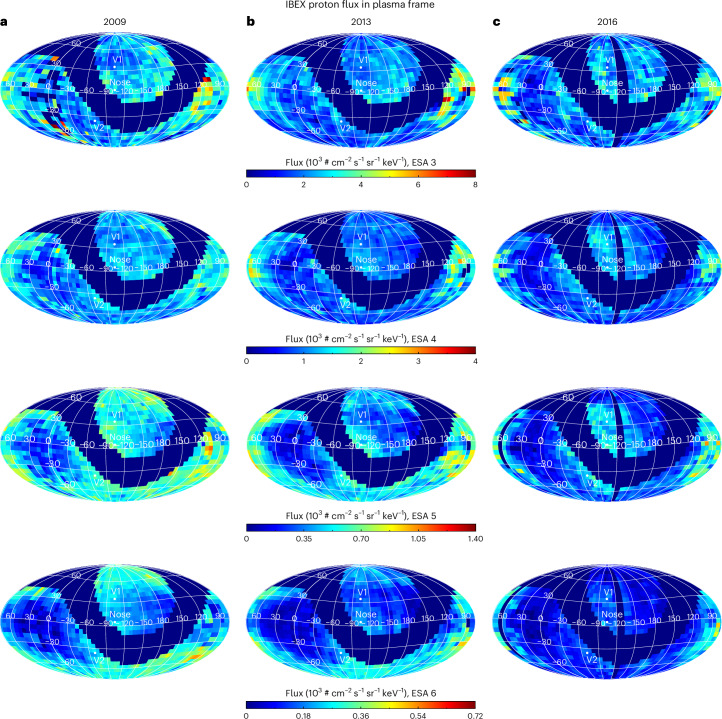
Example sky maps of proton fluxes in the HS plasma frame derived from IBEX-Hi ENA observations. **a**–**c**, We show maps in 2009 (**a**), 2013 (**b**) and 2016 (**c**) for ESAs 3–6, with observations from ref. ^31^. The symbol # refers to the number of protons. Note that pixels near the ribbon are removed. Also note that the proton energy per pixel is different due to the Compton–Getting correction.

The goal of this study is to derive all-sky maps of the HTS compression ratio (that is, its ‘strength’). To do this, we utilize IBEX-Hi observations in the spacecraft ram reference frame and make use of data from 2009–2016 to avoid the large increase in ENA fluxes that occurred starting in late 2016 due to a large increase in SW dynamic pressure. We use IBEX-Hi observations from electrostatic analyser (ESA) steps 3–6 (~1–6 keV) and exclude ESA 2 observations from our analysis, the reason for which is explained in Methods. We use IBEX ENA data transformed to proton fluxes in the HS plasma reference frame from Zirnstein et al.^31^. While in our previous study we developed a method to convert ENA fluxes in the spacecraft frame to proton fluxes in the simulated HS plasma frame, in the current study we use this new proton flux data to compare IBEX data with simulations of test particle acceleration across the HTS and the resulting downstream distributions. Our global minimization scheme normalizes the simulated spectrum to the observations before their comparison (see ‘Finding the best-fit compression ratio at the HTS’ section in Methods). Figure 1 shows several sky maps of IBEX-derived proton fluxes, averaged along the line of sight in the HS plasma frame.

### Upstream SW conditions

We require knowledge of the SW conditions upstream of the HTS to determine the downstream plasma properties corresponding to IBEX observation times (that is, plasma conditions that created ENAs observed later in time by IBEX). This requires propagating SW properties from 1 au to the HTS. In situ observations of SW parameters (for example, density, temperature, speed and magnetic field) collected in the OMNI database from the Advanced Composition Explorer (ACE) and Wind measurements are used, which are observed within ±8° of the ecliptic plane. At higher latitudes, we utilize a model of SW speeds^32^ derived from interplanetary scintillation (IPS) observations^33^. As discussed in some previous studies^33,34^, SW speeds derived from IPS at low latitudes tend to overestimate in situ observations in solar cycle 24. Therefore, a shift is applied to the IPS-derived speeds, separately for each year, down by a value at all latitudes to match OMNI at low latitudes^32^. Whether or not shifting the SW speed by the same value at all latitudes is correct cannot be determined at this stage^35^. Therefore, we include uncertainties in our analysis to account for this unexplained discrepancy (see fig. 24 in ref. ^32^).

Because IPS observations provide only estimates of the SW speed, a reasonable assumption that the SW energy flux is independent of latitude is made^36^, allowing us to use OMNI measurements of speed and densities to derive proton densities at high latitudes^32,34^. The plasma temperature and magnetic field magnitude are also extracted from the OMNI measurements at low latitudes and extrapolated to high latitudes using the following methods. For plasma temperature, we assume that, at SW speeds of 750 km s^−1^, the temperature is 250,000 K based on Ulysses measurements^37,38^ and linearly interpolate from low-latitude OMNI values to high latitudes with this reference point. Due to the variance observed in plasma temperature at high latitudes, we include an uncertainty of 10% at all latitudes. For magnetic field magnitude, we extract the field magnitude from OMNI measurements and apply the Parker spiral equations, yielding radial and tangential field components as a function of latitude. The alpha-to-proton density ratio is assumed to be latitude invariant, which does not noticeably affect our results; thus, we apply OMNI observations of the ratio at all latitudes.

**Fig. 2:**
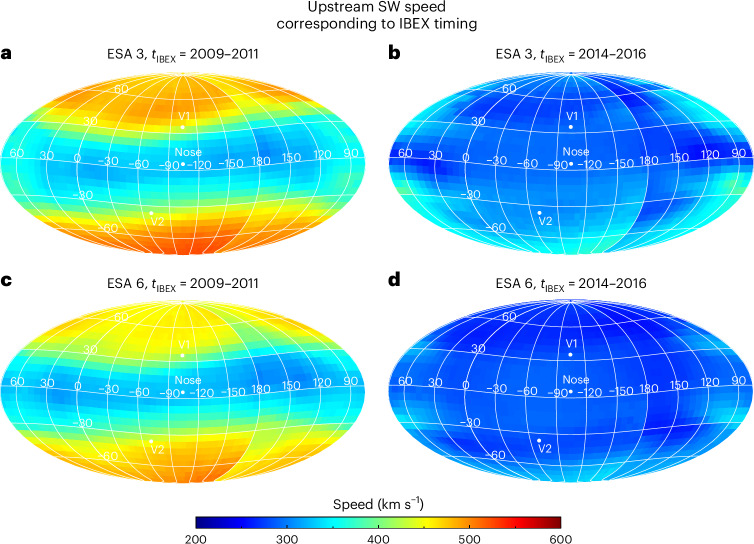
Example sky maps of SW speed upstream of the HTS for two time periods of IBEX observations (2009–2011 and 2014–2016). The maps corresponding to ESA 3 (**a**, **b**) and 6 (**c**, **d**) demonstrate how the ENA time delays are different for each ESA (see also Supplementary Fig. 3). IBEX’s Sun-pointing spinning and the time it takes to observe the entire sky are included.

**Extended Data Fig. 1:**
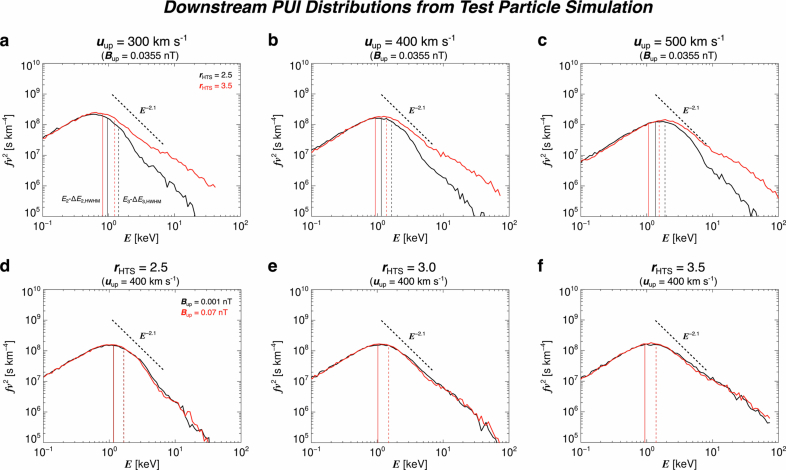
Downstream PUI Distributions from Test Particle Simulation. Examples of downstream PUI distributions from our test particle simulation for different upstream conditions (using case 1 width of ~1 L_R_). (**a**-**c**) We show results for different HTS compression ratios (**r**_HTS_ = 2.5, 3.5) as a function of upstream SW speed (**u**_up_ = 300, 400, 500 km s^−1^). (**d**-**f**) We show results for different upstream magnetic field (**B**_up_ = 0.001, 0.07 nT) as a function of HTS compression ratio (**r**_HTS_ = 2.5, 3, 3.5). The vertical solid lines show the central energy of ESA 2 minus ESA 2’s half width at half maximum. The vertical dashed lines show the same, except for ESA 3. Also shown is a power law with slope of E^−2.1^ for reference. Note that the 2 vertical solid lines and 2 vertical dashed lines in panels d-f overlap each other.

SW parameters at 1 au as a function of latitude and time are propagated using a multi-fluid model to the HTS^12^, providing the plasma conditions upstream of the HTS. Examples of the upstream SW speed for the two IBEX time periods of interest are shown in Fig. 2. Note that the results are a function of the IBEX ESA energy passband because the measurement time at 1 au may be the same, but the travel time for ENAs to get to IBEX from the outer heliosphere is different depending on their energy. The sharp transition in speed at longitude 180° is an effect of IBEX’s annual map-making process^28^. The upstream magnetic field is shown in Supplementary Fig. 4. We note that our modelled magnetic fields do not exactly match those observed by Voyager 1 and 2 (refs. ^23,39^); however, for our purposes, the downstream model proton flux and the upstream magnetic field appear to be weakly dependent on each other (Extended Data Fig. 1d–f).

### Shock microstructure

**Fig. 3:**
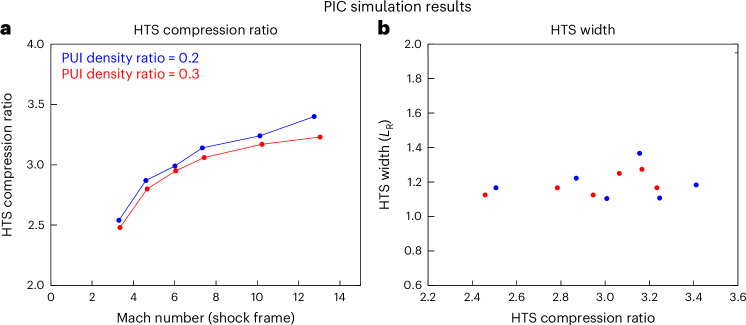
PIC simulation results of the HTS. **a**, PIC simulation results for different upstream Alfvén Mach numbers and PUI density ratios (PUI to total proton density). The resulting compression ratio of the shock depends strongly on the upstream Mach number, but weakly on the PUI density ratio. **b**, PUI foot + ramp width (‘shock width’) as a function of shock compression ratio. The shock width is weakly dependent on the compression ratio (and upstream Mach number). Note that the runs in **b** are the same as those in **a**, colour-coded by their respective PUI density ratio.

PIC simulations are used to determine the relationship between the upstream plasma properties, the shock compression ratio and its microstructure (that is, shock width and overshoot). We ran a set of fully kinetic, two-dimensional PIC simulations^21^ with different upstream Mach numbers, including SW protons, electrons and interstellar H^+^ PUIs, where the PUIs are represented by a generalized filled-shell distribution, extrapolated from SWAP observations^25^, with the cut-off estimated from the upstream SW speed. First, we find that the HTS compression ratio depends only weakly on the PUI density ratio, based on tests assuming ratios of 20% and 30% of the total proton density, for a range of upstream Mach numbers (Fig. 3a). Second, the HTS width (foot + ramp) is nearly invariant of the shock parameters (that is, HTS compression ratio and upstream Mach number; Fig. 3b). Therefore, we simplify our analysis by assuming the HTS width is constant. This, however, may not be true in a highly dynamic system with upstream SW turbulence, as suggested by Voyager 2 observations^23^. Thus, we include the shock width as an uncertainty in our analysis by varying it between 1 ${L}_{R}$ (Fig. 3b) and 1.5 ${L}_{R}$^6^, where ${L}_{R}$ is the upstream advective proton Larmor radius.

### Downstream particle distributions

The PIC simulation results are used to constrain test particle simulations, which we use to simulate the downstream proton distribution for a variety of upstream SW conditions. Realizing the shock compression ratio is minimally affected by the expected range of PUI densities, we ran a set of test particle simulations^6^ while varying upstream SW speed, magnetic field and shock compression ratio (Methods). The shock width is assumed to be constant in the test particle model. Examples of the downstream distributions are shown in Extended Data Fig. 1. We focus on the PUI distributions that dominate the ENA fluxes over most of the IBEX-Hi energy range. Note that a rollover in our test particle results appears below ~1 keV, which is not realistic because the observed ENA spectrum does not show a rollover below ~1 keV. Although it is highly unlikely that core SW protons would fill this gap, it is possible that PUIs injected into the HS plasma by charge exchange could cover this energy range^40^. Because we are concerned only with the distribution immediately downstream of the HTS, we can ignore energies <1 keV and thus exclude IBEX ESA 2 data from our analysis (excluding data below the vertical dashed lines in Extended Data Fig. 1), which overlap the rollover and would not provide realistic results of the ion spectrum. Extended Data Fig. 1 shows that the downstream PUI spectral slope changes with shock compression ratio and upstream SW speed, but not with upstream magnetic field. The PIC simulation shows that the compression ratio does not noticeably change when varying the shock angle values between 75° and 85°. Our MHD simulation shows that the HTS is quasi-perpendicular over most of the sky, except near the north and south heliographic poles in just a few pixels where the shock angle is ~60° or smaller (Supplementary Fig. 1b). Therefore, we caution readers to not rely on our results near the poles (latitudes >|±75°|), where the shock normal angle with respect to the magnetic field reaches ≲60°.

### Compression ratio

**Fig. 4:**
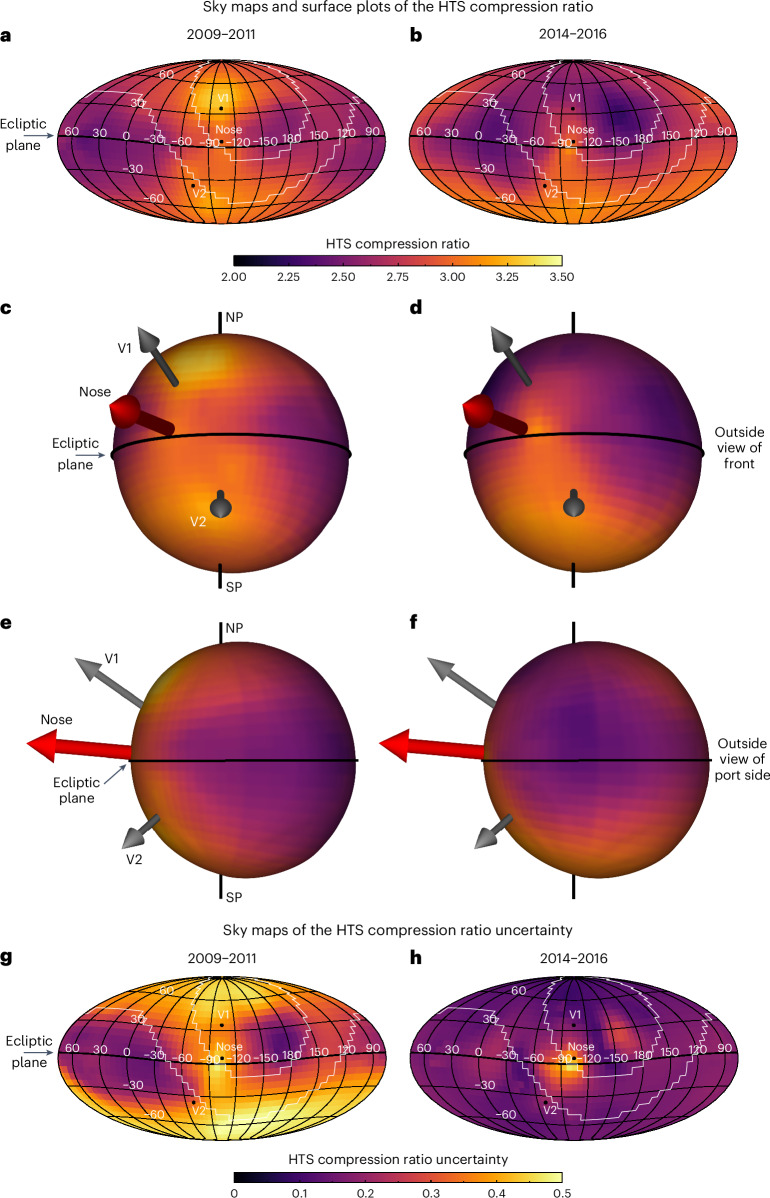
Main results of the HTS compression ratio. **a**,**b**, Sky maps of the HTS compression ratio for IBEX time periods 2009–2011 (**a**) and 2014–2016 (**b**). **c**–**f**, Three-dimensional surface plots of the compression ratio from two viewpoints. The size of the HTS is derived from our MHD simulation, and the colour is the HTS compression ratio value. Shown are views of the front of the heliosphere (offset from the nose) (**c** and **d**) and views of the port side (**e** and **f**). **c** and **e** are from 2009–2011, and **d** and **f** are from 2014–2016. We show arrows for the nose, V1 and V2 trajectories, and lines for the ecliptic plane, the ecliptic north pole (NP) and ecliptic south pole (SP). **g**,**h**, Sky maps of the HTS compression ratio uncertainty for 2009–2011 (**g**) and 2014–2016 (**h**). The white contour in the sky maps shows the region of data surrounding the ribbon excluded from our data input to the minimization process. The high values of the relative uncertainties near the port side of the heliosphere (~15° longitude and ~200° longitude in 2014–2016) are connected to the regularization uncertainty, which result in different levels of smoothness near the local minimum (in compression ratio) for considered variations of the SW parameters. The higher uncertainty in 2009–2011 from the mid-latitude to polar regions is due to the increase in uncertainty from the fast SW speed. The higher uncertainties near the port and starboard lobes in 2014–2016 are due to the regularization minimization procedure attempting to find the best compression ratio in the spatially small, low-compression regions. Smoother transitions in compression across the sky, such as in 2009–2011, yield lower uncertainties after minimization.

Using the information gathered thus far, we perform a global least-squares and minimization with regularization between the model and data-derived proton fluxes to derive the best-fit HTS compression ratio maps. A detailed description of this process is provided in Methods. Figure 4 shows the main results of our work: sky maps and surface plots of the HTS compression ratio, and their uncertainties derived from our analysis. In 2009–2011, solar activity is near the end of solar cycle 23 minimum (accounting for the SW-to-ENA time delay; Supplementary Fig. 3). Thus, the SW speed is typically faster at high latitudes (Fig. 2), yielding higher compression ratios and flatter ENA spectra from the HS. At low-to-mid latitudes, the compression ratio is lower due to the slower SW speeds. The uncertainties are typically <15% for most of the sky except for some small regions, particularly the port side, where the high uncertainties are connected to the regularization uncertainty. There are also high uncertainties in 2009–2011 at high latitudes due to the uncertainty we introduce for the IPS-derived SW speed near 1 au.

Inside the ribbon region, outlined by the white contours, the uncertainties from the minimization process are slightly larger due to the removal of IBEX data (but are typically small). However, because each IBEX pixel is an accumulation of fluxes produced from multiple streamlines connecting to different foot points on the HTS (Supplementary Fig. 7), information surrounding the ribbon gap region is used in the minimization process to fill in the ribbon region.

Interestingly, the port and starboard heliotail lobe regions^41^, roughly located in longitude ranges −30° to 60° and 90° to 180°, respectively, have the smallest compression ratios. This result reflects the steeper proton spectra observed by IBEX. A possible cause of this behaviour, related to the increasing distance to the HTS, is examined in Discussion.

Figure 4b,d,f,h shows the HTS compression ratios and their uncertainties for 2014–2016, which approximately reflects SW conditions produced near solar maximum. The SW speeds in this time frame are slower (by a few hundred kilometres per second), particularly at high positive latitudes, and exhibit a noticeable difference in the northern versus southern hemispheres (Fig. 2). This asymmetry is reflected in the HTS compression ratio primarily because of the relationship between compression ratio and upstream Mach number for quasi-perpendicular shocks^42^.

When comparing Fig. 4a with Fig. 4b (or Fig. 4e,g with Fig. 4f,h), there is a general decrease in compression ratio, mostly occurring at high northern latitudes, while there is little change below the nose and near the tail direction. The uncertainties of the two maps are different, especially at high latitudes. There are smaller uncertainties at high latitudes in 2014–2016 compared with 2009–2011 due to the slower SW speed in 2014–2016 near solar maximum. There is also a high uncertainty near the nose of the heliosphere in both maps due to the higher sensitivity of the compression ratio to the HTS width and interstellar neutral H distribution.

Table 1 shows results in selected directions of the sky. There is no statistically significant change over time in the compression ratio in the nose direction, but there is a statistically significant change near Voyager 1’s direction and a possible change in the direction of Voyager 2. The change at Voyager 1 is probably due to the large change in SW speed in the northern hemisphere as the northern polar coronal hole (PCH) was closing^32,34^. The HTS compression ratio near the starboard lobe moderately decreased over time (but still within 1 − *σ* uncertainties), but no statistically significant change is seen from the port lobe. The north and south poles also show notable differences: the compression ratios are similar in 2009–2011, but the compression ratio in the north pole drops substantially due to the PCH closing (note again that one should be careful interpreting our results at latitudes >|±75°|).

**Table 1:** HTS compression ratio in selected directions of the sky

Direction^a^	2009–2011		2014–2016		Statistically significant change?^e^	Physical causes for observed HTS compression relative to other locations or times
	$\boldsymbol{r}_{\mathbf{{HTS}}}$^b^	${\boldsymbol{\sigma}}_{\mathbf{r}}$	$\boldsymbol{r}_{\mathbf{{HTS}}}$	${\boldsymbol{\sigma}}_{\mathbf{r}}$		
Nose^b^	2.96	0.39	2.85	0.28	No	LISM pressure
Voyager 1	3.25	0.36	2.70	0.17	Yes	PCH–SW properties/evolution
Voyager 2^c^	3.03	0.35	2.96	0.19	No	PCH–SW properties
Port lobe^d^	2.48	0.17	2.51	0.18	No	SW slowing due to distance to HTS
Starboard lobe^d^	2.59	0.19	2.47	0.15	Maybe	SW slowing due to distance to HTS
Central tail	2.61	0.20	2.79	0.17	Maybe	SW slowing due to distance to HTS versus less mass loading due to fewer interstellar neutrals

^a^Directions in ecliptic J2000 (longitude, latitude): Nose = (255.7°, 5.1°) (however, see note b below), Voyager 1 = (256.1°, 35.1°), Voyager 2 = (290.3°, −36.4°), Port lobe = (14.6°, −8.4°), Starboard lobe = (162.6°, 24.9°), North pole = (0°, 90°), South pole = (0°, −90°), Central tail = (75.7°, −5.1°).

^b^Compression ratios and their uncertainties are taken from a single 6° × 6° IBEX pixel nearest to the desired direction. However, due to the higher systematic uncertainties of the regularization routine in smoothing around narrow regions of peaks (that is, near the nose) of the compression ratio, particularly in 2014–2016, we take the nose pixel to be centred at (249°, 9°), shifted one pixel northwards and one pixel starboard. This also moves it away from the ribbon mask region.

^c^It is interesting to note that the simple theoretical formulation for PUI heating across the shock from Shrestha et al.^86^ provides a compression ratio closer to 2.5, although without providing uncertainties. Also, Voyager 2 crossed the HTS multiple times, with compression ratio values of 2.38 ± 0.14 and 1.58 ± 0.71 at TS-2 and TS-3 crossings, respectively^24^, or an average of 1.98 ± 0.36. Thus, our results are inconsistent with the Voyager 2 data in both time periods. The Voyager 1 compression ratio was estimated from magnetic field measurements to be 3.02 ± 0.04 (but ratios in the range 2 and 4 are admissible)^39^, and was estimated to be ${2.6}_{-0.2}^{+0.4}$ from the SW velocity jump^10^. Our compression ratio results vary between 3.25 ± 0.36 and 2.70 ± 0.17, which are roughly consistent with the observations when taking into account their uncertainties.

^d^We take the port and starboard lobe directions to be the lobe centres found by Dayeh et al.^41^. To avoid confusion over the change in time of the lobe centres, we take the arithmetic mean of the centres found by Dayeh et al. in 2009–2011 and 2014–2015, yielding (14.6°, −8.4°) and (162.6°, 24.9°) for the port and starboard lobe centres, respectively.

^e^Statistically significant changes are determined to be ‘yes’ if the compression ratio values observed in 2009–2011 and 2014–2016 lie completely outside their counterpart 1 − *σ* uncertainty range, although there may be overlap of the uncertainty ranges; ‘no’ if the compression ratio values lie close to each other within both uncertainty ranges; and ‘maybe’ if the compression ratios lie just inside their counterpart 1 − *σ* uncertainty range.

## Discussion

By utilizing a sophisticated combination of micro-to-macro modelling tools and global minimization analysis techniques, we have constructed the first all-sky maps of the HTS compression ratio surrounding our solar system. On average over the sky, the HTS compression ratio typically lies between approximately 2.53 and 2.96 in both 2009–2011 and 2014–2016 (±1 − *σ* ranges calculated from the weighted standard deviation of the population), with a mean of ~2.73 over both time periods. Compared with Voyager 2^24^ observations, even when our results’ uncertainties are considered, the model is not consistent with the observations. The individual crossings of the HTS yield an average compression of ~2 (from speed, magnetic field and density measurements). Although the results of our analysis may not strictly be comparable to the timing of Voyager 2’s crossing(s) of the HTS in 2007, partly considering that the compression ratio might change over time and our results are a 3-year average after Voyager 2’s crossing, our model results show almost no change in the compression ratio in the Voyager 2 direction between the two time periods. Also, our methodology does not include an energetic particle (over tens of kilo-electronvolts) precursor, which, if included, may yield a compression ratio closer to that observed by Voyager 2.

**Fig. 5:**
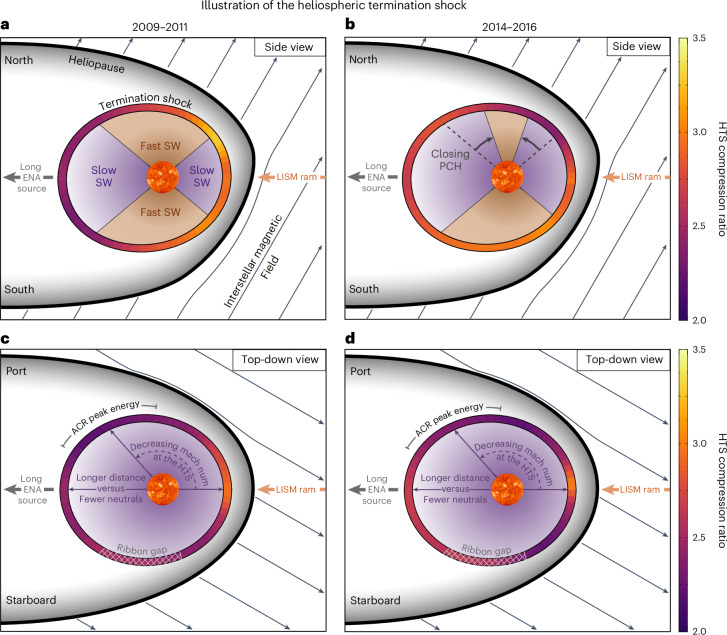
Illustrations of the HTS compression ratio. **a**–**d**, Results are shown in the solar meridional (side view, **a** and **b**) and solar equatorial (top-down view, **c** and **d**) cross-sections through the heliosphere. Also shown are the physical mechanisms responsible for the measured shock compression. The surface of the HTS is colour-coded with the respective HTS compression ratio, for the different time periods (2009–2011, **a** and **c**; 2014–2016, **b** and **d**). Regions and boundaries of the heliosphere are labelled, and the fast versus slow SWs are colour-coded as tan and purple, respectively. The proton density as a function of distance from the Sun is illustrated by the gradients in these colours. The five primary variables controlling the observations are (1) LISM ram pressure, (2) SW speed, (3) closing of the PCH and (4) the amount of SW mass loading with distance to the HTS. Note that the shapes of the heliosphere boundaries in **a** and **b** are adapted from McComas et al.^87^.

Here, we discuss the physical implications of the results, which are illustrated in Fig. 5. First, there are clear latitudinal and longitudinal dependencies observed in the HTS compression ratio. The latitudinal dependence comes primarily from the SW speed, which varies between solar minimum and maximum^38^. Faster SW speeds generally yield stronger shocks; thus, the compression ratio is larger at high latitudes in 2009–2011, reflecting solar minimum conditions. The asymmetric evolution of the SW towards maximum activity in solar cycle 24 is visible in Fig. 4b,f, where the earlier closing of the northern PCH^43^ yielded a smaller compression ratio than in the south.

We propose that the longitudinal dependence is driven by (1) a local maximum of interstellar pressure near the nose of the heliopause^1^, (2) the decrease in the SW number density and bulk SW speed towards the flanks where the HTS distance from the Sun is larger than the nose^44,45^, (3) slowing of the SW by mass loading as the distance to the HTS increases along the flanks and (4) fewer interstellar PUIs in the central tailward direction, leading to a smaller plasma pressure and higher upstream magnetosonic Mach number and, thus, a slightly stronger shock compression^22^.

To explain the minimum compression ratios seen at the tailward flanks of the heliosphere, we look at several possibilities beyond what we stated above. First, using hybrid simulations of the HTS, Giacalone et al.^22^ suggested that particles accelerated to energies >10 keV but <1 MeV, which dominate the particle pressure, are probably similar in pressure across the HTS surface and therefore would not create a spatially dependent shock compression from the nose to the flanks. Another possibility is that these energetic particles reach maximum energies much higher than 1 MeV along the flanks of the HTS (becoming anomalous cosmic rays)^46^, but they do not contribute as much pressure as ~10 keV to <1 MeV particles do, would not be effective at creating an upstream pressure gradient like the one observed by Voyager 2^24,47^ and are probably not responsible for the lower compression at the flanks.

Therefore, we interpret the compression ratio minimum near the flanks as shown in our results to be a consequence of the slowdown of the SW by mass loading. This is because the distance to the shock along the flanks is larger than the distance to the HTS near the nose, effectively decreasing the upstream Mach number and weakening the shock. Another revelation from the results can be seen in the differences in the HTS compression ratio between the north and south poles. In 2014–2016, the HTS near the south pole has a substantially larger compression ratio than the north, which may be caused by faster SW in the south due to the larger PCH, and the disappearance of the PCH in the north, as shown by Fig. 2 for the second time period.

There is no consensus on what heating and acceleration mechanism is primarily operating in the HS. A recent study showed additional heating in the HS may not be needed^48^ to explain the ‘gap’ between modelled and observed ENA fluxes identified in recent years^45,49^. However, it is also believed that particles are probably heated and accelerated by Alfvénic or compressible turbulence in the HS, considering Voyager observations of turbulence in the HS^50^, which probably originated from the transmission of SW turbulence and/or current sheets across the HTS into the HS^51–53^. To demonstrate how an HS heating mechanism may improve the comparisons between modelled and observed ENA fluxes, we have separately simulated ENA maps from a global MHD simulation that includes a potential HS heating mechanism in our methodology based on successful modelling fits to IBEX data^7^. By using our method of velocity diffusion in the HS, the new simulated ENA maps match better to IBEX observations (Supplementary Fig. 8), at least in the forward hemisphere of the heliosphere (except longitudes ~30° to ~120°, which are centred on the heliotail).

Finally, the upcoming Interstellar Mapping and Acceleration Probe (IMAP) mission^54^, which is expected to launch in 2025, will provide ENA measurements with better statistics, over a broader range of energies, and improved temporal cadence. IMAP ENA data will allow us to improve our determination of the HTS compression ratio as a function of space and time and better understand the role of energetic particle mediation on the tailward flanks of the shock with the comprehensive suite of in situ particle instruments onboard the spacecraft. This Article provides a necessary advancement for future studies to improve upon our understanding of the HTS, particularly with IMAP measurements.

## Methods

### Propagating SW and PUI plasma to the HTS

We propagate the SW H^+^, He^++^ and interstellar H^+^ and He^+^ PUI distributions to the HTS using multispecies equations^12^. We solve the following equations coupled by photoionization and charge exchange source terms:

1 $$\frac{1}{{r}^{2}}\frac{{\mathrm{d}}}{{{\mathrm{d}}r}}\left({r}^{2}\rho u\right)=\mathop{\sum }\limits_{i=1}^{4}{S}_{i}^{\rho }$$

2 $$\frac{1}{{r}^{2}}\frac{{\mathrm{d}}}{{{\mathrm{d}}r}}\left({r}^{2}{\rho }_{i}u\right)={S}_{i}^{\rho }$$

3 $$\frac{1}{{r}^{2}}\frac{{\mathrm{d}}}{{{\mathrm{d}}r}}\left({r}^{2}\rho {u}^{2}\right)={S}^{m}-\frac{{{\mathrm{d}}p}}{{{\mathrm{d}}r}}-\frac{{B}_{\varphi }^{2}}{{\mu }_{0}r}-\frac{{B}_{\varphi }}{{\mu }_{0}}\frac{{\mathrm{d}}{B}_{\varphi }}{{{\mathrm{d}}r}}$$

4 $$u\frac{{\mathrm{d}}{p}_{i}}{{{\mathrm{d}}r}}={S}_{i}^{p}-\gamma {p}_{i}\frac{1}{{r}^{2}}\frac{{\mathrm{d}}}{{{\mathrm{d}}r}}\left({r}^{2}u\right)$$

5 $$\begin{array}{c}\frac{1}{{r}^{2}}\frac{{\mathrm{d}}}{{{\mathrm{d}}r}}\left({r}^{2}{B}_{r}\right)=0\\ {B}_{\theta }=0,\\ \frac{1}{r}\frac{{\mathrm{d}}}{{{\mathrm{d}}r}}\left(r{B}_{\varphi }u\right)=0,\end{array}$$

where these equations solve for (1) total mass flux, (2) individual mass fluxes of SW H^+^, He^++^ and interstellar H^+^ and He^+^ PUI separately, (3) total momentum flux, (4) individual internal pressures for each ion species and (5) interplanetary magnetic field with radial (${B}_{r}$) and tangential (${B}_{\varphi }$) components whose total magnitude at 1 au is extracted from OMNI data and initialized at 1 au using the Parker Spiral equations^55^. The angle between the radial direction and the magnetic field direction at 1 au is assumed to be 45°, a typical average angle consistent with OMNI data. The source terms (${S}^{\rho },{S}^{m},{S}^{p}$) on the right side of equations (1)–(4) can be found in the methods section of our previous study^12^. The source terms include the effects of photoionization and charge exchange, using a three-dimensional spatial distribution for neutral H derived from our global MHD model, including the recent update to neutral H density from SWAP^56,57^, and for interstellar He derived from the ‘cold’ model^58,59^.

The global MHD model simulates the SW–LISM interaction by solving the single fluid MHD equations that are then coupled, through source terms, to neutral H atoms that are solved using Boltzmann’s equation. The inner SW boundary conditions (defined at 1 au and extrapolated to the inner boundary of the simulation at 10 au) of the global MHD model are extracted from the OMNI database and Ulysses observations, averaged over 2004–2009, which is chosen on the basis of the expected average return time for ENAs measured in 2009–2016^31^. In the low-latitude, slow SW, the inner boundary conditions are extracted from the OMNI database: speed 449 km s^−1^, density 6.53 cm^−3^ (for both protons and alphas as a single fluid), temperature 1.05 × 10^5^ K and radial magnetic field 37.4 μG (assuming a Parker spiral). In the high-latitude, fast SW, the inner boundary conditions are extracted from Ulysses data taken during its third fast polar scan in 2007: speed 743 km s^−1^, density 2.23 cm^−3^ (both protons and alphas), temperature 2.98 × 10^5^ K and radial magnetic field 34.7 μG. The latitude of separation between the slow and fast SW is |±37°|, based on Ulysses observations^38^. The inner boundary conditions are then extrapolated to 10 au assuming a Parker spiral for magnetic field and adiabatic expansion of the plasma from 1 to 10 au from the Sun.

The outer boundary conditions of the MHD model, set at 1,000 au from the Sun, are extracted from IBEX and New Horizons’ SWAP observations: speed 25.4 km s^−1^ (ref. ^60^), total effective plasma density 0.09 cm^−3^ (interstellar H^+^ density is ~60% or 0.054 cm^−3^, and the remainder is interstellar He^+^ with 10% of the density, 0.009 cm^−3^ (ref. ^61^), but 40% of the dynamic pressure; note that interstellar neutral He^+^ is not solved as a separate fluid, but rather that in the MHD/Boltzmann charge exchange source terms the plasma is considered to be composed of this H^+^ and He^+^ mixture, thus approximating the presence of interstellar He^+^ through charge exchange), neutral H density 0.17 cm^−3^, temperature 7,500 K (ref. ^60^) and magnetic field strength 2.93 μG and orientation (227.28°, 34.62°)^62^. The neutral H density is chosen such that the filtration of interstellar neutral H through the front of the heliosphere yields H densities consistent with New Horizons’ SWAP observations^56^.

We propagate the SW/PUI parameters from 1 au to the HTS, where the HTS location is assumed to be that from the same global MHD simulation used to produce the neutral H density distribution^12^ (Supplementary Fig. 1a), but we scale up the distances by ~10% to match the averaged observed distances in the Voyager 1 and 2 directions. Because the HTS location is variable over time, and its distance is uncertain, we propagate a 1 − *σ* uncertainty of 5% to the distance to the HTS in our analysis (see ‘Finding the best-fit compression ratio at the HTS’ section). Once the SW/PUI parameters upstream of the HTS are found, we use these to run a range of test particle simulations with different upstream parameters encompassing the results from solving equations (1)–(5). The details of the test particle simulations are described in the ‘Deriving model proton fluxes downstream of the HTS’ section. We note that the study by McComas et al.^63^ found four points in the sky from Voyager 1 and 2 crossing the HTS and the disconnection of magnetic field lines from the HTS. To fit the points, they assumed the simplest solution—an offset sphere. While our HTS shape has features qualitatively similar to McComas et al.^63^, such as the closest point being slightly below the nose and the farthest point in the heliotail direction, our MHD-modelled HTS does not match the shape of an offset sphere. Rather, it is a non-spherical, blunt-shaped HTS. Because McComas et al.^63^ do not estimate the uncertainty of their final result, it is difficult to tell if their results are statistically consistent with ours.

Moreover, we note that our MHD simulation overestimates the thickness of the HS by ~15 au in the Voyager 1 and 2 directions as compared with the Voyagers’ observations. However, the distances to the middle of the HS in the Voyager 1 and 2 directions are, on average, consistent with Voyager 1 and 2 observations (that is, the HTS distance is smaller than observed, and the HP distance is larger than observed). Thus, in terms of current capabilities, this is a reasonable simulation to use for our analysis, particularly for the flow streamlines and SW-ENA time delays.

For simplicity in our analysis, we create two sky maps of the HTS compression ratio, one corresponding to IBEX data taken in years 2009 through 2011, and another for 2014 through 2016. These time periods approximately correspond to solar minimum and maximum conditions in solar cycle 24, respectively (accounting for the time delay for changes to occur in ENAs observed at 1 au), and the combination of three annual maps per period improves the statistics of the analysis. The time delay between SW outflow measured at 1 au and IBEX measurements at 1 au can range anywhere between ~1.5 and >7 years, depending on the ENA energy and direction in the sky. We describe ways of handling this complexity in ‘Estimating time delays between SW and ENA measurements’ in Supplementary Information.

We solve equations (1)–(5) starting at 1 au using SW conditions for each year from 1998.5 to 2016.5 (the IPS SW speed model has one solution per year^32^) to cover the possible time delays between SW and ENA detection yielded by the simulation. This yields SW and PUI plasma properties upstream of the HTS for a large range of years after propagation to the HTS. Example plots of SW speed, density and magnetic field upstream of the HTS are shown in Supplementary Fig. 2.

### Deriving model proton fluxes downstream of the HTS

With a set of plasma conditions upstream of the HTS for a range of years (see ‘Propagating SW and PUI plasma to the HTS’ section), and the total time delay between SW and ENA measurements at 1 au (see ‘Estimating time delays between SW and ENA measurements’ in Supplementary Information), we subtract the total time delay in the IBEX spacecraft ram frame for each year in the two time periods and find the upstream SW properties corresponding to the ENA measurements. We then average the plasma properties over time that correspond to the two IBEX time periods. Examples of the time-averaged upstream plasma conditions are shown in Fig. 2 and Supplementary Fig. 4. One can see the effects of the spacecraft’s Sun-pointed spinning and the abrupt change in plasma conditions at longitude 180° because of the disjoint in time while using only ram frame observations.

Using this information, we know that the ranges of upstream magnetic field magnitude and SW speed are [0.001, 0.07] nT and [200, 600] km s^−1^, respectively. We do not need to know the upstream plasma density, because our analysis uses only normalized downstream fluxes. We also assume that the PUI-to-SW proton number density ratio is 0.25 everywhere, based on their small effect on the shock structure and compression ratio as seen in PIC simulation results^21^, shown in Fig. 3. As one can see, the compression ratio of the simulated HTS depends only weakly on the PUI density ratio and is small compared with the uncertainties of our results; therefore, for simplicity we ignore its effects on our analysis. The PUI-to-SW proton number density ratio does change moderately across the sky. Estimates from, for example, Zirnstein et al.^45^, show that the ratio can change between ~0.2 and 0.3 (see their fig. 2), with the lowest ratio near the nose of the heliosphere and the highest between the poles/flanks and tail of the heliosphere. We will relax this assumption of a constant density ratio in a future study.

To derive model proton fluxes downstream of the HTS, we use a test particle simulation^6^ over the range of parameters found above, where we assume the upstream velocity distribution is a filled shell with adiabatic cooling index set to 2.9, extrapolated from New Horizons’ SWAP observations halfway to the HTS^25^. The test particle simulation solves the transport of PUIs across a mean field shock structure plus a turbulence component whose power ratio is consistent with Voyager 2 observations. Unlike in Zirnstein et al.^6^, here we do not assume an arbitrary amount of enhanced turbulence near the PUI foot of the shock. The test particle simulation solves the Lorentz force equation for the total magnetic field (mean field plus turbulent field), with a convective electric field term and a cross-shock potential electric field term^64^. As shown by Zirnstein et al.^6^, the majority of >1 keV PUIs undergo one or more reflections under shock drift acceleration that forms the PUI tail downstream of the shock.

We run the test particle simulation for all combinations of three variables: (1) upstream magnetic field magnitude (assuming a perpendicular shock) with values [0.001, 0.0355, 0.07] nT; (2) upstream SW speed with values [200, 300, 400, 500, 600] km s^−1^; and (3) HTS compression ratio with values [2.0, 2.5, 3.0, 3.5, 4.0]. Examples of the downstream distribution results are shown in Extended Data Fig. 1. We do not run test particle simulations for different SW or PUI densities because these just scale the downstream distribution by constant factors, and our derivation of the optimal compression ratio involves minimizing chi-square between normalized IBEX and model proton fluxes (see ‘Finding the best-fit compression ratio at the HTS’ section). The downstream distribution is linearly interpolated between the values shown above for different upstream SW conditions.

Extended Data Fig. 1 shows how the downstream PUI distribution function depends on the upstream magnetic field, SW speed and HTS compression ratio. For example, Extended Data Fig. 1a demonstrates how the distribution becomes flatter at energies above ~1 keV and higher in intensity. As the upstream SW speed increases for a single compression ratio (Fig. 4a–c), the distribution shifts towards higher energies because the downstream flow is moving faster away from a solar inertial observer (or, for example, IBEX). Moreover, as the upstream SW speed increases, the compression ratio increases and acceleration from the cross shock electric field is stronger. Also notice that in each panel we plot several solid and dashed vertical lines. The solid vertical lines show the central energy of ESA 2 (~0.7 keV) minus ESA 2’s half-width at half-maximum for each downstream distribution curve. The dashed vertical lines are similar, except they correspond to ESA 3 minus its half-width at half-maximum. We show these lower ‘edges’ of the ESA passbands because they are close to the rollover in the PUI distribution. We do not include core SW protons in our test particle simulation because they are not responsible for ENA production above ~0.7 keV due to their low temperature^24,65^. We also exclude ESA 2 data from our analysis owing to the rollover in the PUI distribution, whose energies overlap where the rollover occurs, and IBEX data do not show a rollover below 1 keV.

Figure 4d–f shows how the downstream PUI distribution depends on the upstream magnetic field, for different HTS compression ratios in each panel. Clearly, there is no visible difference in the results as a function of upstream magnetic field. This is because, for a single HTS compression ratio and upstream SW speed, the gain in energy per particle depends on the ratio of the mean turbulent magnetic field to the mean field power, that is, ${\left(\updelta B/B\right)}^{2}$, which is a constant in our model. We note that ${\left(\updelta B/B\right)}^{2}$~0.16 downstream of the shock (or ${\left(\updelta B/B\right)}^{2}$~0.01 at *kR*_g_ = 1, similar to Voyager 2 measurements; note that *B* changes with the different upstream parameters that we simulate). Using a constant ${\left(\updelta B/B\right)}^{2}$ is, however, a simplifying assumption. In future work, it may be more reasonable for ${\left(\updelta B/B\right)}^{2}$ to depend on distance from the Sun (or the time it takes for the SW to travel from the Sun to the HTS) in different parts of the sky, in the same manner as we do to scale the diffusion coefficient as defined in equation (7).

Finally, we compare our test particle results with Voyager 2’s Low Energy Charged Particle (LECP) observations, as shown in Supplementary Fig. 5. We set the compression ratio to 2.5 (approximately the average of Voyager 2’s observations and our model results, although we note that they do not overlap even with their 1 − *σ* uncertainties), and show results for upstream flow speeds of 300 km s^−1^ and 400 km s^−1^, which is the range of speeds that Voyager 2 observed within ~0.7 au of the HTS. With the lack of statistics at energies above ~15 keV in our test particle model, it is difficult to compare with the LECP data. Our results appear to be within a factor of ~5–10 of the data but cannot seem to match both data points at the same time. This also implies the slope of our model is not the same as the data. We also compare our results with Cassini-INCA proton flux data in Supplementary Fig. 5. While the intensities are roughly within less than an order of magnitude of the data, and sometimes within the data uncertainties, our model does not appear to develop slopes similar to the INCA proton flux data, as shown by, for example, Dialynas et al.^17^.

### Effects of velocity diffusion, adiabatic heating and charge exchange in the HS

Because the IBEX proton fluxes represent the line of sight-averaged flux in the HS for each IBEX ESA, we must ‘undo’ or deconvolve the effects of propagation through the HS to properly compare the observed proton fluxes with the modelled proton fluxes just downstream of the HTS. We do this by solving the Parker transport equation with charge exchange source terms on the large-scale plasma flows obtained from our global heliosphere model, similar to our previous work^7,66^, but this time in all directions of the sky. Each line of sight has ENA emissions produced at different distances through the HS, with each having a plasma flow streamline connected back to a different point on the HTS. Therefore, for each of these streamlines, first we trace the flow back to the HTS via a second-order Runge–Kutta (midpoint) method. Upon reaching the HTS, we initialize a downstream proton distribution that is, on average, close to what is expected downstream of the HTS based on prior studies (that is, a kappa distribution^31,48^ with a kappa index of 2.2; the kappa index is varied between 2 and 2.4 to estimate the 1 − *σ* uncertainty of the index in the final results). While it would make more sense to have a spatially varying kappa index downstream of the HTS, currently it is not possible to determine what they should be immediately downstream of the shock. Therefore, instead, we take a nominal value for all locations on the HTS and test different kappa indices that are then propagated as uncertainties. We note that using kappa indices of 2 and 2.4 do not change the compression ratio results noticeably. Then, we solve the Parker transport equation with source terms, forward in time to the ENA emission position, as shown below in finite difference form (see Zirnstein et al.^7^ for more details)

6 $$\begin{array}{c}{f}_{j}^{\;n+1}={f}_{j}^{\;n}+\frac{\Delta s{\rm{e}}^{-3{w}_{j}}}{{u}_{p}{\Delta w}^{2}}\left[{D}_{j+\frac{1}{2}}{\rm{e}}^{{w}_{j+\frac{1}{2}}}\left({f}_{j+1}^{\;n}-{f}_{j}^{\;n}\right)-{D}_{j-\frac{1}{2}}{\rm{e}}^{{w}_{j-\frac{1}{2}}}\left({f}_{j}^{\;n}-{f}_{j-1}^{\;n}\right)\right]\\ +\frac{\Delta s}{3{u}_{p}}\left(\nabla \cdot {\mathbf{u}}\right)\left[\frac{{f}_{j+1}^{\;n}-{f}_{j-1}^{\;n}}{2\Delta w}\right]+\frac{\Delta s}{{u}_{p}}\left[{\eta }_{j}^{n}{f}_{{\rm{H}},\;j}^{\;n}-{\beta }_{j}^{n}{f}_{j}^{\;n}\right]\end{array},$$

where $w=\mathrm{ln}\left(v\right)$ and $\Delta s$ is the step size along the flow streamline. The second term on the right-hand side is velocity diffusion, with diffusion coefficient $D\left(v\right)={D}_{0}{v}^{\alpha }$ (where ${D}_{0}$ is the diffusion amplitude and $\alpha$ is the spectral index), the next is flow divergence (causing adiabatic heating/cooling) where $\left(\nabla \cdot {\mathbf{u}}\right)$ is calculated using the global MHD simulation’s change in density compression along flow streamlines, and the last is the charge exchange source term. This gives us a final proton distribution/flux at the ENA emission point along the line of sight in question. Note that (1) the typo in equation 11 in Zirnstein et al.^7^ with the missing $\Delta s$ in the charge exchange source term is fixed here, and (2) we have simplified the production of protons by separating out the neutral H distribution ${f}_{\rm{H}}$ and the charge exchange production rate $\eta$ in equation 2 of Zirnstein et al.^7^. This does not affect our results because new protons produced in the HS are at injected at speeds in the plasma frame with energies around ~100 eV. Equation (6) is solved using a ‘forward-time, central-difference’ method. Therefore, the step size along the streamline, $\Delta s$, must be sufficiently small to maintain stability. We have found that a step size of ≤0.02 au is sufficient. The range of speeds over which we solve equation (6) is 1 to 6,200 km s^−1^, or ~0.005 eV to 200 keV, in natural log-space, with 150 bins.

We calculate the ratio of the final distribution function over the initial distribution just downstream of the shock at a specific particle speed $v$, that is, $F\left(v\right)={f}_{f}\left(v\right)/{f}_{i}\left(v\right)$, for each streamline (where $v$ is the desired ENA speed to measure and the initial distribution ${f}_{i}\left(v\right)$ is described by a kappa = 2.2 distribution). Each streamline ratio is averaged along the line of sight to find the ‘best’ average change in the distribution. Next, we divide the IBEX proton flux along the line of sight by this ratio (see equations (12) and (13)), effectively undoing the effects of velocity diffusion, adiabatic heating and charge exchange as the distribution evolves through the HS. This is possible because the flux is proportional to the distribution. Examples of this ratio across the sky are shown in Supplementary Fig. 7. We note that taking an average of this ratio along the line of sight may introduce unknown systematic uncertainties; however, our current methods are the best that we can provide at this time.

The diffusion coefficient, $D\left(v\right)={D}_{0}{v}^{\alpha }$, is based on a previous study^7^ where the IBEX-Lo and IBEX-Hi spectra were fit by chi-square minimization to find the best values for ${D}_{0}$ and $\alpha$. By fitting a parabola to the minimum of each spectral index case in the left panel of fig. 4 from Zirnstein et al.^7^, we find the best-fit values with minimum chi-square are ${D}_{0}=8.18\times {10}^{-9}\,\mathrm{km}^{2}\,\mathrm{s}^{-3}$, and $\alpha =1.31\pm 0.20$, in the central tail direction. The uncertainties of the diffusion amplitude ${D}_{0}$ and the spectral index $\alpha$ are not independent but reflect their tight linear correlation in log space (see the narrow parabolas in the left panel of fig. 4 from Zirnstein et al.^7^). Therefore, they need to be considered jointly as one uncertainty. We parameterized this uncertainty by the $\alpha$ uncertainty, and for the 1 − *σ* uncertainty of $\alpha$ we use the corresponding ${D}_{0}$ value based on the correlation between the parameters. See the ‘Finding the best-fit compression ratio at the HTS’ section for information on the propagated uncertainties. We note that, while this diffusion coefficient was derived from a single direction in the sky, the amplitude of ${D}_{0}$ changes with direction in the sky. The spectral index $\alpha$ = 1.31 is, interestingly, halfway between the diffusion index for incompressible/Alfvén turbulence and compressible/wave-like turbulence^67^, both of which are observed in the HS^50^.

Because our diffusion coefficient was derived only in the central tail direction, we make a few scaling modifications to apply it to other directions in the sky. First, we set the nominal $\alpha$ = 1.31 across the sky, because we include its uncertainty in our analysis. Second, we set ${D}_{0}=8.18\times {10}^{-9}\,\mathrm{km}^{2}\,\mathrm{s}^{-3}$ as the nominal value in the central tail pixel but scaled by a certain factor. This scaling factor accounts for the fact that the heliosphere simulation used to derive the diffusion coefficient in our prior study has different SW and VLISM properties, yielding a different distance to the HTS in the central tail direction. One might set the scaling factor to the inverse square of the distance to the shock^68^ (which is approximately proportional to the ratio of turbulence to mean field power, ${\left(\updelta B/B\right)}^{2}$) with respect to that in the central tail direction. This means that directions where the HTS is closer to the Sun would have a higher level of turbulence because of less time spent to reach the shock (and less time for the dissipation of turbulence). The scaling factor in this case would be ${\left({r}_{1{{\mathrm{au}}}}/r\left(\varOmega \right)\right)}^{\omega }$ where $\varOmega$ signifies any particular direction in the sky and $\omega =9(1+\varGamma )/4$ is the power law spectral index (see equation 35 in Zank et al.^68^). However, this formulation works only for a single inner boundary SW speed. Because we have different speeds as a function of longitude (because of PUI mass loading) and latitude, we update this ratio to be a function of SW propagation time to the HTS rather than distance, yielding ${\left({\tau }_{{{\mathrm{tail}}}}/\tau \left(\varOmega \right)\right)}^{\omega }$. In this way we have replaced ${r}_{1{{\mathrm{au}}}}$ with the time for travel to the HTS in the central tail direction, ${\tau }_{{{\mathrm{tail}}}}$. Thus, ${D}_{0}$ in the central tail pixel direction is scaled by a factor ${ \sim \left({\tau }_{{{\mathrm{tail}}},{{\mathrm{ref}}}}/{\tau }_{{{\mathrm{tail}}}}\right)}^{\omega }$, where ${\tau }_{{{\mathrm{tail}}},{{\mathrm{ref}}}}=1.34\,{\mathrm{years}}$ is the reference mean time from Zirnstein et al.^7^ where the diffusion coefficient was originally derived, and ${\tau }_{{{\mathrm{tail}}}}=1.19\,{\mathrm{years}}$ is the mean time in the heliosphere simulation used in this paper. The scaling index $\omega \cong$ 2–3, where $\omega$ is closer to 3 within and near the ionization cavity, and $\omega$ is closer to 2 farther beyond the ionization cavity^68^. By estimating the mean spectral index of the evolution of Voyager 1, Voyager 2 and Pioneer 11 observations of ${\left(\updelta B/B\right)}^{2}$, shown in fig. 4 in Zank et al.^68^, we find $\omega \cong2.5$ is a good approximation from ~20–40 au, making the first scaling factor ${\left({\tau }_{{{\mathrm{tail}}},{{\mathrm{ref}}}}/{\tau }_{{{\mathrm{tail}}}}\right)}^{2.5}$. These results are also quite similar to the results obtained from a more recent, sophisticated model of turbulence transport^69^.

We then must apply a second scaling factor for ${D}_{0}$. This factor is based on the time it takes the SW to reach the HTS in directions of the sky other than the central tail direction in the current simulation (because the distance to the HTS and SW speed is not the same in all directions). Thus the second scaling factor is ${\left({\tau }_{{{\mathrm{tail}}}}/\tau \left(\varOmega \right)\right)}^{2.5}$, where $\tau \left(\varOmega \right)=\left\langle r\left(\varOmega \right)/{u}_{p}\left(\varOmega \right)\right\rangle$ is the mean time it takes the SW to reach the HTS in a certain direction of the sky, and ${\tau }_{{{\mathrm{tail}}}}$ is the mean time in the central tail direction. Thus, the faster the SW speed (${u}_{p}\left(\varOmega \right)$) and/or closer distance to the HTS ($r\left(\varOmega \right)$), the larger this ratio is, and the larger the turbulence power.

Finally, the total scaled diffusion amplitude, ${D}_{0}^{{\prime} }\left(\varOmega \right)$, can be written as

7 $${D}_{0}^{{\prime} }\left(\varOmega \right)={D}_{0}{\left(\frac{{\tau }_{{{\mathrm{tail}}},{{\mathrm{ref}}}}}{{\tau }_{{{\mathrm{tail}}}}}\right)}^{2.5}\times {\left(\frac{{\tau }_{{{\mathrm{tail}}}}}{\tau \left(\varOmega \right)}\right)}^{2.5}={D}_{0}{\left({\tau }_{{{\mathrm{tail}}},{{\mathrm{ref}}}}/\tau \left(\varOmega \right)\right)}^{2.5},$$

where ${D}_{0}^{{\prime} }\left(\varOmega \right)$ replaces ${D}_{0}$ in equation (6).

We note that most global heliosphere models of IBEX ENA measurements underestimate the intensities by a factor of ~2–3 (refs. ^45,49,70^), either globally or in certain regions of the sky. Because these models usually include only adiabatic heating effects, it suggests that further particle heating may be occurring in the HS, which could explain this discrepancy. Some studies have suggested that particle heating and acceleration by reconnection^71–73^, turbulence^67,74^ or shocks^75^ may also occur in the HS. Currently there is no consensus on which mechanism, or mechanisms, may dominate over others, and how to implement these in a global heliosphere model. Heating by shocks passing through the HS, however, would not affect our analysis because this primarily raises the intensity of ENAs in the IBEX-Hi energy range, but does not noticeably change the spectral slope^75^. Other mechanisms that primarily accelerate particles producing suprathermal tails is beyond the energy range considered here. Finally, a recent study^48^ demonstrated that particle acceleration at the HTS based on test particle simulations may be sufficient to explain the IBEX-Hi ENA spectrum without the need for additional, non-adiabatic heating in the HS. However, that determination was made only for IBEX ENA data in the Voyager 2 direction and therefore is limited in its conclusions regarding global heating in the HS.

In our study, we include a velocity diffusion process of particles as they travel along the HS plasma flow streamlines, with a velocity diffusion coefficient based on previous fits to IBEX data^7^. The results of the prior study suggested that the spectral index of the diffusion coefficient lies between acceleration by Alfvénic turbulence ($\alpha$ = 2/3) and compressible turbulence ($\alpha$ = 2)^67^, that is, close to the value of 1.3 that we use through the HS, as noted before. However, the spectral index has an uncertainty (see ‘Finding the best-fit compression ratio at the HTS’ section), which is propagated through our results. As shown in Supplementary Fig. 8, the inclusion of velocity diffusion greatly improves the comparison with IBEX GDF-separated data. The old simulation results (Supplementary Fig. 8a) greatly underestimate the observations (Supplementary Fig. 8c–e) by more than a factor of 2. By including velocity diffusion in the HS, the new simulation results (Supplementary Fig. 8b) are increased by approximately a factor of 2 or more. We note that the high flux from heliotail at mid to high latitudes (except the central downwind direction near the ecliptic plane) go beyond 100 flux units. This is primarily due to the steady-state nature of the MHD simulation used to create these maps, where consistent fast SW flows down the tail. In reality, it would be a mixture of slow to fast SW^45^.

### Compton–Getting correction for realistic HS flow frame

It is important to note that the IBEX proton fluxes are in the simulated, steady-state HS plasma frame^31^. The upstream SW conditions and downstream flow speeds may, therefore, be different than what we expect on the basis of ACE/wind and IPS observations. Moreover, when performing our analysis, we must vary the shock compression ratio for all combinations of upstream SW speeds—thus, the IBEX proton fluxes are not likely to be in the correct reference frame at each iteration of the chi-square minimization routine. Therefore, we calculate Compton–Getting correction factors for the IBEX proton fluxes for each direction in the sky, ESA, time period and compression ratio tested in our analysis. These correction factors are multiplied to the IBEX proton fluxes and energies in the minimization routine for the appropriate variable value. The correction factor is calculated as

8 $$c\left(\varOmega ,{E}_{p},t,{r}_{{{\mathrm{HTS}}}}\right)=1-2\frac{\Delta u}{{v}_{p}}\cos \left[\left\langle \beta \left(\varOmega ,{E}_{p}\right)\right\rangle \right]+{\left(\frac{\Delta u}{{v}_{p}}\right)}^{2},$$

where $c$ is the correction factor as a function of direction in the sky ($\varOmega$), central energy of the ESA in the simulated plasma frame (${E}_{p}$), time period *t* and shock compression ratio ${r}_{{{\mathrm{HTS}}}}$. The particle speed in the simulated HS plasma flow frame is ${v}_{p}=\sqrt{2{E}_{p}/{m}_{{\mathrm{H}}}}$. The variable $\Delta u$ is the speed difference between the MHD simulation’s HS plasma frame and the desired frame based on the SW speed upstream of the HTS and the HTS shock compression ratio. We estimate this as

9 $$\Delta u\left(\varOmega ,{E}_{p},t,{r}_{{{\mathrm{HTS}}}}\right)=\frac{{u}_{{{\mathrm{SW}}},u}\left(\varOmega ,{E}_{p},t\right)}{{r}_{{{\mathrm{HTS}}}}}-\left\langle {u}_{{{\mathrm{MHD}}},d}\left(\varOmega ,{E}_{p}\right)\right\rangle,$$

where ${u}_{{{\mathrm{SW}}},u}\left(\varOmega ,{E}_{p},t\right)$ is the SW speed upstream of the HTS, and $\left\langle {u}_{{{\mathrm{MHD}}},d}\left(\varOmega ,{E}_{p}\right)\right\rangle$ is the SW speed immediately downstream of the HTS from the MHD simulation. We note that the downstream speed from the MHD simulation is averaged over all streamlines that connect back to the HTS from different radial increments along the IBEX line of sight direction, $\varOmega$, and weight averaged along the line of sight, using similar averaging methodology as Zirnstein et al.^31^. Equation (9) simplifies the problem by assuming that the differences in flow speed do not influence the plasma flow patterns in the HS. This effectively assumes that the two different plasma frames are parallel (when $\Delta u > 0$) or anti-parallel (when $\Delta u < 0$).

Because the two reference frames for the transformation are parallel (or anti-parallel), the angle $\beta$ can be calculated as the angle between the proton velocity in the MHD-simulated HS plasma frame, ${{\boldsymbol{v}}}_{p}$, directed towards IBEX, and the simulated HS plasma frame velocity, ${{\mathbf{u}}}_{{{\mathrm{MHD}}}}$, such that

10 $$\begin{array}{c}\beta \left(r,\varOmega ,{E}_{p}\right)={\cos }^{-1}\left[\frac{{{\mathbf{v}}}_{p}\cdot {{\mathbf{u}}}_{{{\mathrm{MHD}}}}}{\left|{{\mathbf{v}}}_{p}\right|\left|{{\mathbf{u}}}_{{{\mathrm{MHD}}}}\right|}\right]\\ \left\langle \beta \left(\varOmega ,{E}_{p}\right)\right\rangle =\frac{{\int }_{{r}_{{{\mathrm{HTS}}}}}^{{r}_{{{\mathrm{HP}}}}}\beta \left(r,\varOmega ,{v}_{p}\right)\omega \left(r,\varOmega ,{v}_{p}\right){{\mathrm{d}}r}}{{\int }_{{r}_{{{\mathrm{HTS}}}}}^{{r}_{{{\mathrm{HP}}}}}\omega \left(r,\varOmega ,{v}_{p}\right){{\mathrm{d}}r}}\end{array}.$$

Angle $\beta$ is calculated at every position along each IBEX line of sight and then weight averaged to produce $\left\langle \beta \right\rangle$.

Supplementary Figs. 9 and 10 show examples of the Compton–Getting correction factor for the all-sky maps at ESA 3 and 6 and ${r}_{{{\mathrm{HTS}}}}$ = 2.5 and 3.5. The most distinct feature in all panels is that the correction factor is <1 at high latitudes, especially for larger ${r}_{{{\mathrm{HTS}}}}$ and for the second time period (2014–2016). The reason for this is that the (steady-state) MHD simulation assumed fast SW speeds at 1 au of 743 km s^−1^ at latitudes >|±37°|, whereas the speeds derived from IPS-derived models^32^ are slightly lower (note that IPS speeds are shifted down at all latitudes to match OMNI). This effectively means that the plasma frame is moving too fast away from the observer in the simulation, and therefore IBEX-derived proton fluxes and corresponding energies need to be decreased to compensate. The reason why this is more pronounced in 2014–2016 is that the solar cycle is approaching solar maximum in the time-delayed ENA source frame and, thus, the average SW speeds at high latitudes are much smaller than the steady-state, solar minimum-like simulation.

The second distinct feature in these maps is that the correction factor is sometimes above 1 at lower latitudes, particularly near the nose and tail. It becomes less than 1 for large ${r}_{{{\mathrm{HTS}}}}$, similar to the reasons stated above. The correction factors above 1 at low latitudes suggest either the MHD-simulated SW speeds were too low and/or the MHD-simulated compression ratio was too high (compared with the value assumed in the calculation of each specific map). Overall, by performing these corrections, we aim to partially remove the influence of the MHD simulation’s assumptions for SW speed and HTS compression ratio on our results. We tested the sensitivity of our compression ratio results when using these correction factors versus not using them and found no statistically significant change compared with the total uncertainties of our results.

### Finding the best-fit compression ratio at the HTS

To find the best-fit HTS compression ratios as a function of direction in the sky, we first start with the proton distribution in the HS averaged over the line of sights derived from IBEX observations. Note that the source lengths of the IBEX data are considered to be constant at 30 au, as reported by Zirnstein et al.^31^ who only needed to know the slope of the spectrum and thus assumed the source length was 30 au in all directions of the sky. Here, we do not change the source length used to calculate the IBEX data because we ‘normalize’ the IBEX and model proton spectra to each other before computing the compression ratios. Therefore, setting a constant source length is sufficient, reasonably assuming the source lengths of ENAs from ~1.1 to 4.3 keV are similar^44^. See more information on this normalization factor below.

Let ${j}_{t,p,s}\pm \updelta {j}_{t,p,s}$ denote the derived flux and its uncertainty for year $t$, pixel $p$ and ESA step $s$. While the fluxes are derived for each observed ESA step, the energy in the plasma frame ${E}_{t,p,s}$ differs from the nominal central energy in the heliocentric frame owing to the Compton–Getting effect^76–78^. For simplicity, we enumerate pixels using a single integer $p=\mathrm{1,2},\ldots ,\mathrm{1,800}$. In this study, we accumulate the fluxes over several years corresponding to time period $\tau$ (that is, 2009–2011 and 2014–2016). In general, we calculate the energy and the average flux over this period as well as its uncertainty as follows:

11 $${\bar{E}}_{\tau ,p,s}=\frac{1}{\left|\tau \right|}\sum _{t\in \tau }{E}_{t,p,s}$$

12 $${\bar{j}}_{\tau ,p,s}=\frac{1}{\left|\tau \right|}\sum _{t\in \tau }{j}_{t,p,s}/{F}_{t,p,s}$$

13 $$\updelta {\bar{j}}_{\tau ,p,s}=\frac{1}{\left|\tau \right|}{\left(\sum _{t\in \tau }{\delta j}_{t,p,s}^{2}/{F}_{t,p,s}^{2}\right)}^{\frac{1}{2}},$$

where $|\tau |$ denotes the number of years included in the sum. We omit missing data points in these sums. As mentioned earlier, the IBEX fluxes and their uncertainties are divided by another scaling factor, the distribution ratio that includes the effects of velocity diffusion, adiabatic heating and charge exchange, which we define here as ${F}_{t,p,s}$.

The proton spectrum observed along each line of sight includes contributions from multiple streamline foot points at the HTS. The contribution along line of sight $p$ from point $q$ on the HTS in ESA step $s$ is denoted as ${m}_{s,p,q}$. Note that the lines of sight are numbered with index $p$, while points at the HTS are numbered with $q$. Because we are interested only in relative contributions, we normalize the weights using the following formula:

14 $${\widetilde{m}}_{s,p,q}=\frac{{m}_{s,p,q}}{{\sum }_{q=1}^{1800}{m}_{s,p,q}}.$$

This formula ensures that the weights for a given line of sight sum up to 1.

In our analysis, we compare the derived proton fluxes from IBEX with modelled fluxes as a function of compression ratio. Let ${g}_{\tau ,q,s}(E,R)$ denote the modelled proton flux at point $q$ at the HTS, in energy step $s$, for conditions corresponding to period $\tau$ at energy $E$ and compression ratio $R$. As stated earlier, a relative Compton–Getting factor ${c}_{\tau ,p,s}(R)$ needs to be applied to the derived flux and corresponding energy in each line of sight (for details, see ‘Compton–Getting correction for realistic HS flow frame’). These correction factors are multiplied to the average flux and energy in equations (15) and (16).

Based on the above, we want to minimize the following least-squares expression:

15 $${\chi }_{{\rm{LS}},\tau }^{2}\left({\mathbf{R}},{\mathbf{a}}\right)=\sum _{s}\sum _{p}\frac{{\left({c}_{\tau ,p,s}\left({R}_{p}^{{\prime} }\right){\bar{j}}_{\tau ,p,s}-{a}_{p}{\sum }_{q}{\widetilde{m}}_{s,p,q}{g}_{\tau ,q,s}\left({{c}_{\tau ,p,s}\left({R}_{p}^{{\prime} }\right)\bar{E}}_{\tau ,p,s}{R}_{q}\right)\right)}^{2}}{{\left({c}_{\tau ,p,s}\left({R}_{p}^{{\prime} }\right)\updelta {\bar{j}}_{\tau ,p,s}\right)}^{2}}.$$

It can be rewritten in an equivalent form:

16 $${\chi }_{{\rm{LS}},\tau }^{2}\left({\mathbf{R}},{\mathbf{a}}\right)=\sum _{s}\sum _{p}\frac{{\left({\bar{j}}_{\tau ,p,s}-{c}_{\tau ,p,s}^{-1}\left({R}_{p}^{{\prime} }\right){a}_{p}{\sum }_{q}{\widetilde{m}}_{s,p,q}{g}_{\tau ,q,s}\left({{c}_{\tau ,p,s}\left({R}_{p}^{{\prime} }\right)\bar{E}}_{\tau ,p,s},{R}_{q}\right)\right)}^{2}}{{\left(\updelta {\bar{j}}_{\tau ,p,s}\right)}^{2}}.$$

In the above expression, we seek the best-fit compression ratio and normalization factor vectors: ${\mathbf{R}}={\left\{{R}_{q}\right\}}_{q=1,\ldots ,\mathrm{1,800}},{\mathbf{a}}={\left\{{a}_{p}\right\}}_{p=1,\ldots ,\mathrm{1,800}}$. Note that the IBEX proton flux maps exclude pixels affected by the IBEX ribbon flux (Fig. 1). Therefore, we remove them from the sum in equation (16). Consequently, the normalization factors for these pixels ${a}_{p}$ are not constrained in our study and remain undefined. They are, however, not the primary interest of this study, and thus we do not attempt to estimate these values. We note that McComas et al.^79^ have released the first IBEX team-validated ribbon/GDF separation scheme and the separated maps. This differs from our simple exclusion of the ribbon pixels from our analysis, which is a reasonable assumption for our purposes.

The Compton–Getting correction in equation (16) depends on the effective compression ratio in each pixel, which we calculate according to the following formula:

17 $${R}_{p}^{{\prime} }=\mathop{\sum }\limits_{q}{\widetilde{m}}_{s,p,q}{R}_{q}.$$

However, for numerical reasons, minimization in the general form given in equation (16) with this substitution would be too complicated. In our minimization scheme, we instead use an iterative procedure to obtain subsequent estimations of the compression ratio vector, and we use the result from the previous iteration in equation (17) in the next iteration. The Compton–Getting corrections are calculated for compression ratios 2.0, 2.5, 3.0, 3.5 and 4.0. Between these values, we use linear interpolation to get the value of ${c}_{\tau ,p,s}\left({R}_{p}^{{\prime} }\right)$. We denote the values at the calculated compression ratios as ${c}_{\tau ,p,s,r}.$

As the model flux is calculated for a finite number of energy bins and compression ratio values, we use a two-step interpolation scheme. The model is calculated for 66 logarithmically spaced energies $e$ from 0.1 keV to 15 keV and for the same compression ratio values as the Compton–Getting correction. Let ${\widetilde{g}}_{\tau ,q,s,e,r}$ denote the model value for calculated energy $e$ and compression ratio $r$. Because, for each HTS position, the energy grid is the same, we find the two nearest logarithms of the energy bins $\log {e}_{1}$ and $\log {e}_{2}$ to $\log ({{c}_{\tau ,p,s}\left({R}_{p}^{{\prime} }\right)\bar{E}}_{\tau ,p,s})$. With these two, we define the following transformation tensor:

18 $${{\bf{T}}}_{\tau ,p,s,e,r}=\left\{\begin{array}{cc}\frac{\log {e}_{1}-\log \left({{c}_{\tau ,p,s}\left({R}_{p}^{{\prime} }\right)\bar{E}}_{\tau ,p,s}\right)}{\log {e}_{1}-\log {e}_{2}} & {\rm{if}}\,{e}={e}_{2}\\ \frac{\log {e}_{2}-\log \left({{c}_{\tau ,p,s}\left({R}_{p}^{{\prime} }\right)\bar{E}}_{\tau ,p,s}\right)}{\log {e}_{2}-\log {e}_{1}} & {\rm{if}}\,{e}={e}_{1}\\ 0 & {\rm{otherwise}}\end{array}\right.$$

Using this tensor, we interpolate over the compression ratio using the following formula:

19 $$\begin{array}{c}{g}_{\tau ,q,s}\left({{c}_{\tau ,p,s}\left({R}_{p}^{{\prime} }\right)\bar{E}}_{\tau ,p,s},{R}_{q}\right)=\mathop{\sum }\limits_{e}{{\bf{T}}}_{\tau ,p,s,e,{r}_{q}^{0}}{\widetilde{g}}_{\tau ,q,s,e,{r}_{q}^{0}}+\\ \left({R}_{q}-{r}_{q}^{0}\right)\frac{{\sum }_{e}{{\bf{T}}}_{\tau ,p,s,e,{r}_{q}^{+}}{\widetilde{g}}_{\tau ,q,s,e,{r}_{q}^{+}}-{\sum }_{e}{{\bf{T}}}_{\tau ,p,s,e,{r}_{q}^{-}}{\widetilde{g}}_{\tau ,q,s,e,{r}_{q}^{-}}}{{r}_{q}^{+}-{r}_{q}^{-}}\end{array},$$

where ${r}_{q}^{0},{r}_{q}^{+},{r}_{q}^{-}$ denote the closest, closest among larger, and closest among smaller values (${r}_{q}^{0}={r}_{q}^{+}$ or ${r}_{q}^{0}={r}_{q}^{-}$) from the above set to the ${R}_{q}$ value obtained from the previous iteration within available compression ratios.

The minimization of equation (16) is, in most situations, ill-conditioned because of the high number of fit parameters. Consequently, regularization is needed to minimize equation (16). We use the Tikhonov regularization method^80,81^ with the following regularization term:

20 $${\chi }_{{\rm{reg}}}^{2}=\sum _{q}\sum _{{q}^{{\prime} }\in N\left(q\right)}\frac{{\left({R}_{q}-{R}_{{q}^{{\prime} }}\right)}^{2}}{{\rm{dist}}{\left(q,{q}^{{\prime} }\right)}^{2}},$$

where $N(q)$ gives 8 pixels around pixel $q$, and ${\rm{dist}}\left(q,{q}^{{\prime} }\right)$ is the angular distance between the centres of a pair of pixels. We limit the sum to the nearest neighbours for computational reasons.

In our fitting, we minimize the following sum:

21 $${\chi }^{2}(\lambda )={\chi }_{{\rm{LS}},\tau }^{2}+\lambda {\chi }_{{\rm{reg}}}^{2},$$

where $\lambda$ is the regularization parameter. We find the optimal regularization parameter using the L-curve technique^82^. Namely, we minimize equation (21) as a function of $\lambda$, and we inspect the trajectory of the optimal ($\log {\chi }_{{\rm{LS}},\tau }^{2},\log {\chi }_{{\rm{reg}}}^{2})$. The curve made by this trajectory has an L shape (Supplementary Fig. 11a,c), and we seek the corner where the curvature of the trajectory is the largest (Supplementary Fig. 11b,d). The solution found in the corner is adopted as our best fit. Note that, while ${\chi }_{{\rm{LS}},\tau }^{2}$ does not vary as much as ${\chi }_{{\rm{reg}}}^{2}$, compression ratio maps produced from the right-most edges of the L curve are overly and unrealistically smoothed owing to a large regularization parameter.

The interpolations discussed previously are initially spanned between compression ratios 2.5 and 3.0 for each point at the HTS. After obtaining the best fit, we span the interpolations in subsequent iterations between the two nearest values to the compression ratio determined in the previous iteration separately for each direction. We then continue to iterate until the problem converges. While we find there is a small set of pixels in which the compression ratio is close to one of the values used for the modelling, and the best-fit values oscillate between values slightly above and below this value (for example, slightly above 3.0 and then slightly below 3.0), the range of these oscillations is much smaller than the derived uncertainties.

To calculate the uncertainty of the derived compression ratio, we start with the uncertainty related to the IBEX data uncertainty. This uncertainty can be calculated from the inverted matrix of second derivatives around the minimum of equation (21) calculated for the optimal ${\lambda }_{{\rm{opt}}}$:

22 $${\bf{V}}=\frac{{\chi }_{{\rm{LS}},\tau ,\min }^{2}}{{n}_{{\rm{dof}}}}{\left(\frac{1}{2}\frac{{\partial }^{2}{\chi }_{\min }^{2}\left({\lambda }_{{{\mathrm{opt}}}}\right)}{\partial {\mathbf{R}}\partial {\mathbf{R}}}\right)}^{-1}.$$

Square roots of the diagonal terms of this matrix give the uncertainties for each pixel and time period, ${\sigma }_{\tau ,p}$. The uncertainty matrix is multiplied by the reduced ${\chi }_{{\rm{LS}}}^{2}$. The number of degrees of freedom is generally given as [(number of ESA steps used in the fit) − 1] × (number of pixels with the data) − 1,800.

Next, we check how the fit compression ratios respond to uncertainties in the (1) SW speed at 1 au, (2) density of interstellar neutral hydrogen in the heliosphere, (3) SW-to-ENA time delay, (4) HTS microstructure used in our test particle model, (5) distance to the HTS, (6) core SW temperature at 1 au, (7) the diffusion coefficient spectral index and (8) the initial kappa index of the proton distribution just downstream of the HTS. For this, the calculations are repeated with modified modelled proton fluxes and Compton–Getting correction factors corresponding to these changes, where we add or subtract 1 − *σ* values to each variable. The 1 − *σ* uncertainties are assumed to be: (1) −10% for SW speed^32^, where we choose to subtract uncertainties to mimic the shifting of IPS-derived SW speeds to match OMNI data at low latitudes (during these time periods, the IPS-derived SW speeds tend to overestimate the OMNI data). We note that ~10% of the IPS-derived SW speeds during these two time periods is the maximum shift required to match in situ observations of the SW near the ecliptic plane (fig. 11 in ref. ^32^).

As a side note, although we should take half of the maximum shift to approximate the 1 − *σ* uncertainty (that is, half of 10%), we do not because there may be additional uncertainty in the SW speed in how it effects the normalization factor that comes out of the global minimization results (see equation (16)). Ideally, the normalization factor should be ~1 across the sky if our test particle model was accurate in all aspects; however, we end up with values between 2 and 4 at low to mid-latitudes, closer to 1 at high latitudes and >6 near the heliotail. Part of the reason for this is that we keep the upstream SW density constant in our test particle model because the normalization of the model to the data is part of our minimization routine; thus, scaling by a density should not noticeably change the results. In fact, the latitudinal density profile of the SW near solar minimum, for example, has higher densities at lower latitudes and lower densities at high latitudes. Because we assume in the test particle model that the upstream total density is set to 0.00133 cm^−3^ (or the core SW proton density is 0.001 cm^−3^), this is possibly too small at low latitudes and too large at high latitudes. By correcting for this, the normalization factor may indeed be improved. To test the sensitivity of the compression ratio results on the density, we have scaled the test particle fluxes by factors of 0.5, 1, 2 and 4, and the minimized compression ratio results practically the same. Another possible reason for the normalization factor results could be that the IBEX proton flux data were derived assuming the HS thickness in all directions is 30 au (see Zirnstein et al.^31^). This is probably what causes the normalization factor to be >6 in the downwind hemisphere. Finally, the SW speed (that is, Mach number) of the shock can affect the downstream test particle fluxes and their slope. It is unclear how our methods may be negatively affecting the results; it may be due to the lack of temporal resolution better than 1 year, or the shifting of the IPS SW speeds at all latitudes down to match OMNI data in the ecliptic plane. Therefore, we consider 10% (instead of 5%) to be a reasonable 1 − *σ* uncertainty for the SW speed, propagated through to the final compression ratio results.

The additional uncertainties are as follows: (2) −10% for interstellar neutral H density^56^, where we subtract by 1 − *σ* owing to the historical value of interstellar H density^83^ being lower than the currently accepted value^56^; (3) +33% for SW-to-ENA time delay as a rough estimate for uncertainty (no preference for adding or subtracting 1 − *σ*), to account for uncertainties related to using a steady-state MHD simulation’s flow streamlines in the HS; (4) changing the HTS foot width to 1.5 ${L}_{R}$, based on uncertainties in the Voyager 2 shock speed^6,23^, as our estimate of 1 − σ uncertainty; (5) −5% for the distance to the HTS, ${l}_{{{\mathrm{HTS}}}}$, where we choose to subtract 1 − *σ* to mimic the effects of increasing the SW speed (that is, a closer HTS means less time slowed down by mass loading; we choose a ±5% uncertainty, which is approximately ±5 au in the nose-ward hemisphere, based on how the HTS may move over time informed by dynamic models^84^ and observations^12^); (6) +10% for the core SW temperature at 1 au, particularly because of the lack of data at high latitudes without Ulysses (we choose 10% because it approximately reflects the variation in high-latitude SW speed variations^37,38^); (7) ±0.2 for the diffusion coefficient spectral index, where we take half of the summed differences from the nominal case as the uncertainty for $\alpha$; and (8) ±0.2 for the kappa index, chosen based on the fact that the majority of data lie between kappa ~2 and 2.4 (with kappa ~2 close to the mode of the all-sky distribution and highly skewed to larger values) based on analyses of IBEX data^18^ and their agreement with test particle results^6,48^. The uncertainty is also calculated using half of the summed differences from the nominal case, that is, taking their average.

We find the final, best-fit compression ratio per time period and pixel, ${R}_{\tau ,p}^{f}$, shown in Fig. 4a,b. The total uncertainty of the compression ratio is given as

23 $$\begin{array}{c}{\sigma }_{{{\mathrm{tot}}},\tau ,p}=\left\{{\sigma }_{\tau ,p}^{2}+{\left[{R}_{\tau ,p}^{\;f}-{R}_{\tau ,p}(u-{\sigma }_{u})\right]}^{2}\right.\\ +{\left[{R}_{\tau ,p}^{\;f}-{R}_{\tau ,p}\left({n}_{H}+{\sigma }_{n}\right)\right]}^{2}+{\left[{R}_{\tau ,p}^{\;f}-{R}_{\tau ,p}\left(t+{\sigma }_{t}\right)\right]}^{2}\\ \begin{array}{c}+{\left[{R}_{\tau ,p}^{\;f}-{R}_{\tau ,p}\left({L}_{{{\mathrm{HTS}}}}+{\sigma }_{L}\right)\right]}^{2}+{[{R}_{\tau ,p}^{\;f}-{R}_{\tau ,p}({d}_{{{\mathrm{HTS}}}}-{\sigma }_{d})]}^{2}\\ \begin{array}{c}+{\left[{R}_{\tau ,p}^{\;f}-{R}_{\tau ,p}\left(T+{\sigma }_{T}\right)\right]}^{2}\\ +{\left[\frac{1}{2}\left(\left|{R}_{\tau ,p}^{\;f}-{R}_{\tau ,p}\left(\alpha +{\sigma }_{\alpha }\right)\right|+\left|{R}_{\tau ,p}^{\;f}-{R}_{\tau ,p}\left(\alpha -{\sigma }_{\alpha }\right)\right|\right)\right]}^{2}\end{array}\\\left.+{\left[\frac{1}{2}\left(\left|{R}_{\tau ,p}^{\;f}-{R}_{\tau ,p}\left(\kappa +{\sigma }_{\kappa }\right)\right|+\left|{R}_{\tau ,p}^{\;f}-{R}_{\tau ,p}\left(\kappa -{\sigma }_{\kappa }\right)\right|\right)\right]}^{2}\right\}^{1/2},\end{array}\end{array}$$

where the first term is the propagated statistical uncertainty of ${R}_{\tau ,p}^{\;f}$. The second term contains a factor of 1/4, which combines the averaging and halving to obtain the desired (5%) 1 − σ uncertainty. Note that the terms in parentheses in equation (23) represent parameters of ${R}_{\tau ,p}$. The maps of the total uncertainties are shown in Fig. 4c,d.

## Supplementary information

Supplementary Information Supplementary Figs. 1–11, Supplementary Discussion of Figs. 3 and 6 and Supplementary References.

Supplementary Data 1 Compression ratio data in Fig. 4a.

Supplementary Data 2 Uncertainty data in Fig. 4g.

Supplementary Data 3 Compression ratio data in Fig. 4b.

Supplementary Data 4 Uncertainty data in Fig. 4h.

## Data Availability

The results reported in this study shown in Fig. 4 are provided with this Article, as Supplementary Data 1–4.

## Code availability

Code algorithms used to produce results of this study are available from the corresponding author upon reasonable request. Code used to perform the global chi-square minimization analysis is publicly available online via Zenodo at 10.5281/zenodo.16321848 (ref. ^85^).
