# Supplementary material for: Global heliospheric termination shock strength in the solar–interstellar interaction
Source: Nat Astron. 2025 Aug 19;9(10):1495–510. doi: 10.1038/s41550-025-02634-3 (PMC12534181; doi:10.1038/s41550-025-02634-3)
Supplement: Supplementary file 1 — Supplementary Figs. 1–11, Supplementary Discussion of Figs. 3 and 6 and Supplementary References. [file 41550_2025_2634_MOESM1_ESM.pdf]

nature astronomy

Supplementary information https://doi.org/10.1038/s41550-025-02634-3

Global heliospheric termination shock strength in the solar–interstellar interaction

In the format provided by the authors and unedited

Supplementary Information

Table of Contents

Supplementary Figure 1	Page 2
Supplementary Figure 2	Page 3
Supplementary Figure 3	Page 4
Estimating Time Delays between SW and ENA Measurements	Page 4
Supplementary Figure 4	Page 5
Supplementary Figure 5	Page 6
Supplementary Figure 6	Page 6
Mapping Positions on the HTS to each IBEX Pixel	Page 6
Supplementary Figure 7	Page 7
Supplementary Figure 8	Page 7
Supplementary Figure 9	Page 8
Supplementary Figure 10	Page 8
Supplementary Figure 11	Page 9
References	Page 9

Here we provide supplemental figures referenced in the main text and Methods section of the paper, along with a few descriptions.

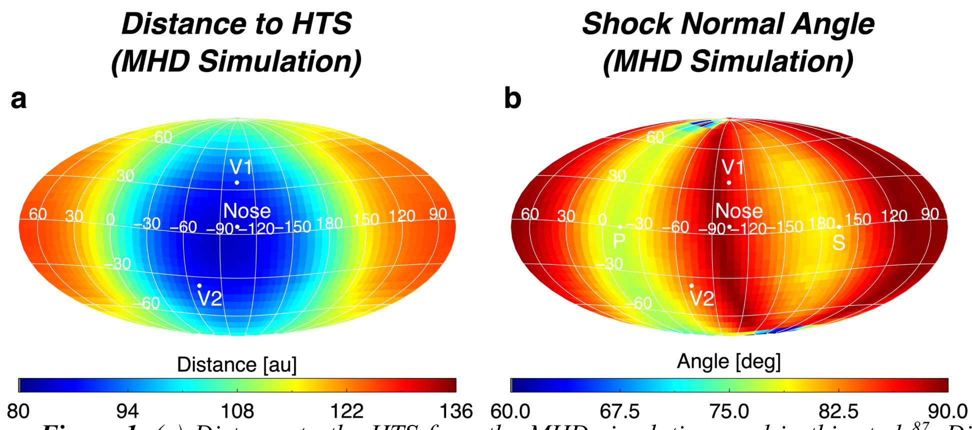

Supplementary Figure 1. *(a) Distance to the HTS from the MHD simulation used in this study[87]. Distances were scaled such that the average distance to the HTS from the simulation in the V1 and V2 directions is the same as the average of the observed crossings from the Voyager spacecraft[88,89]. (b) Shock normal angle from the MHD simulation. For most of the sky, the shock normal angle is ≳75°, except very close to the solar heliographic poles (tilted by 7.25° from the ecliptic poles). Both maps are plotted in ecliptic J2000 coordinates.*

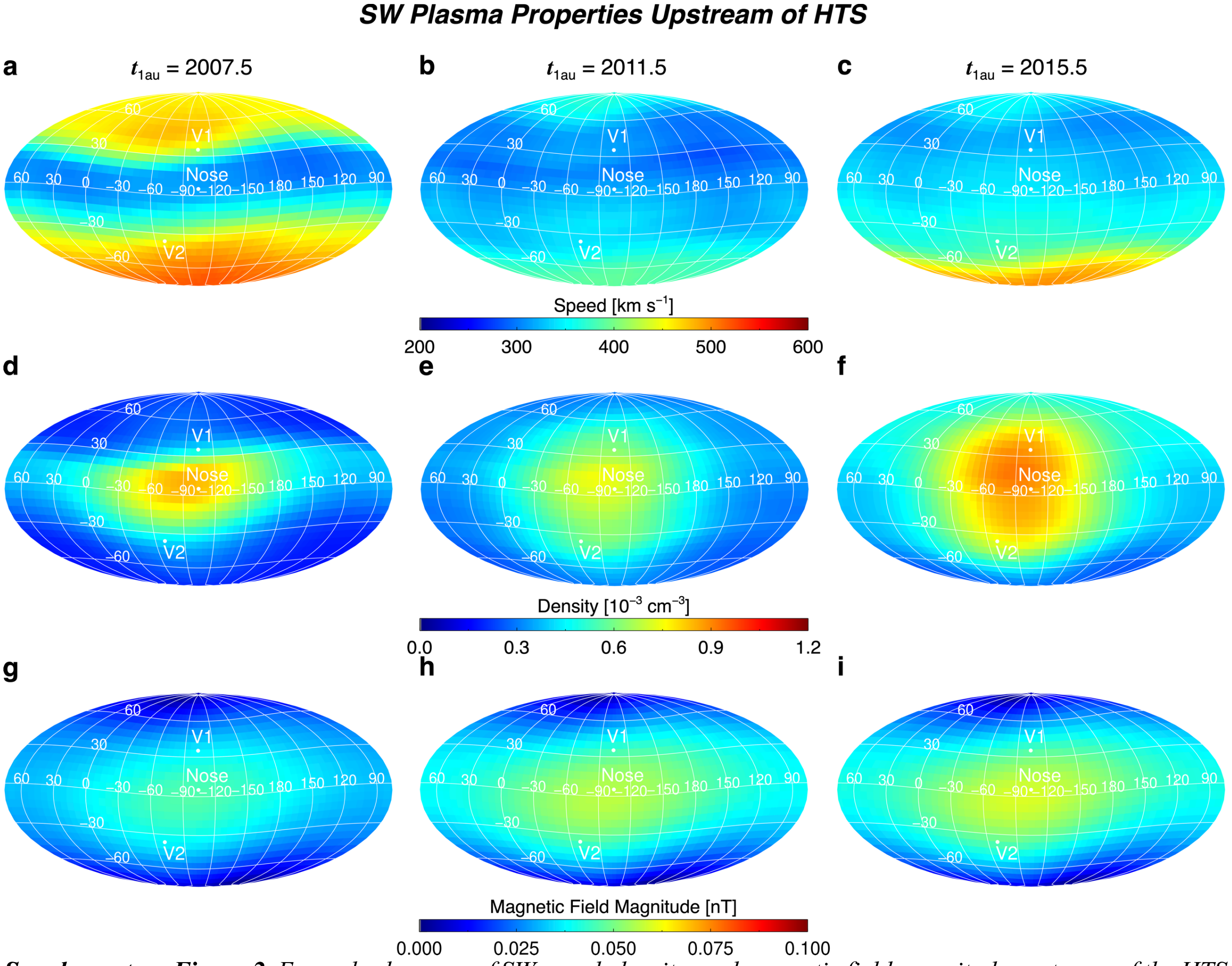

Supplementary Figure 2. *Example sky maps of SW speed, density, and magnetic field magnitude upstream of the HTS derived from IPS/OMNI data taken at 1 au in (a) 2007.5, (b) 2011.5, and (c) 2015.5 and propagated to the HTS using Eq. (1)-(5). Note that the results for* t_{1au} *= 2015.5 reflect the enhancement in SW pressure beginning at the Sun in late 2014, but we do not use this data in our analysis. We restrict our analysis to times before IBEX first observed an increase in ENA fluxes associated with the SW pressure increase.*

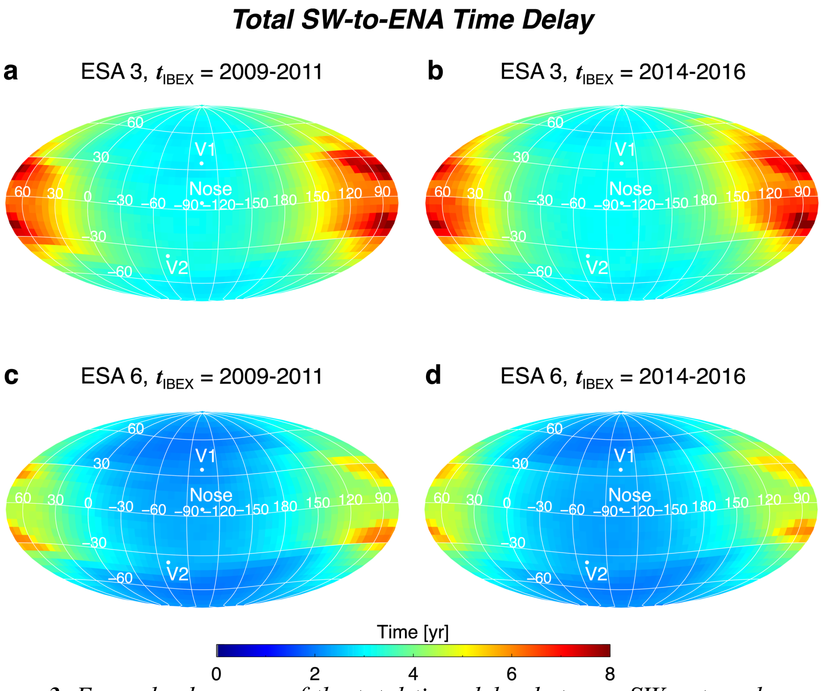

Supplementary Figure 3. *Example sky maps of the total time delay between SW outward propagation at 1 au and IBEX ENA observation, for ESA 3 (a, b) and ESA 6 (c, d). These results are based on flow streamlines from a steady-state simulation of the heliosphere[87], but speeds based on the IPS/OMNI model[90]. See text for details.*

Estimating Time Delays between SW and ENA Measurements

A better way than assuming a single delay time for all ENA energies and pixels in the sky is to first use a global MHD simulation to estimate the total time delay per pixel and ESA passband of IBEX. Therefore, we utilize a global MHD simulation[91], which includes the higher neutral H density found from SWAP observations[92] and adapts the interstellar plasma density to include the presence of interstellar He^+ in the charge exchange source terms. Moreover, this simulation also constrains the interstellar densities to require that the middle of the simulated HS averaged in the Voyager 1 and 2 directions is the same as the middle of the HS averaged over Voyager 1 and 2 observations. The interstellar magnetic field at the outer boundary of the simulation is the same found by fitting to the IBEX ribbon[93]. Using this simulation, for each pixel in the sky, we integrate backwards the time it takes an ENA at a specific energy within an IBEX ESA passband to go back to its origin in the HS (first starting immediately downstream of the HTS), for the HS plasma at that point to flow back to the HTS, and for the supersonic SW to propagate from that position back to 1 au. Note that because the observed ENA signal is a line-of-sight integration, we calculate multiple delay times by assuming the ENA may come from anywhere in the HS along the line of sight, tracing back to the HTS and Sun at multiple foot points, yielding multiple delay times. We then find the mean delay time by averaging these delay times weighted by the local proton flux in the HS, i.e., higher proton fluxes producing higher ENA emissions yields a larger weight. This method of weighting is the same as that used by Zirnstein et al.[87], see specifically their Eq. (10)-(11). As shown by Zirnstein et al.[87], the local proton flux, $f_p v^2/m_p$, is derived as a local weight from the line of sight integrated ENA flux equation in order to calculate the ENA and proton flux in the HS plasma frame per pixel in the sky (see Equations (4)-(11) in Section 2.2 of their paper for more details). We then repeat this procedure over a range of ENA energies covering the IBEX

ESA passband, and weight-average them using the ESA response function[94]. This is repeated for each ESA of IBEX-Hi (except ESA 2). Example sky maps of the total delay times for ESAs 3 and 6 are shown in Supplementary Figure 3.

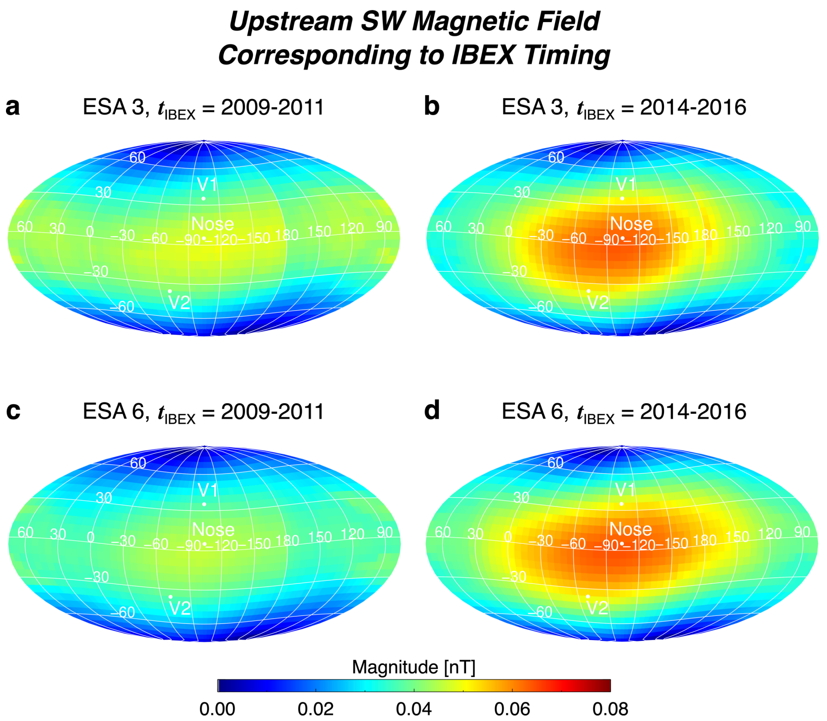

Supplementary Figure 4. *Similar to Figure 2 in the main text, except we show magnetic field magnitude. Note that the magnetic field magnitude we obtain in our SW propagation model does not exactly match that observed by Voyager 1 and 2 upstream of the HTS.*

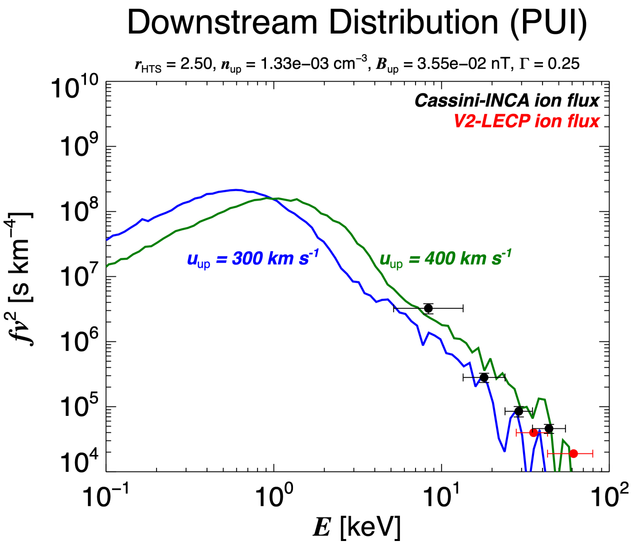

Supplementary Figure 5. *Comparison of our test particle results (blue and green curves) downstream of the HTS to Cassini-INCA data converted to ion fluxes[95] from 2009-2012 (black dots with error bars) and Voyager 2 LECP data[96] taken downstream of the HTS and averaged over DOY 248-252 in 2007 (red dots with error bars). Note that the test particle model has low statistics at energies above ~15 keV. Here, the PUI density ratio is Γ = 0.25, same as in Figure 4 in the main text. We assume the compression ratio is 2.5, approximately halfway between the Voyager 2 observation (~2) and our compression ratio results (~3). They do not overlap within 1-σ uncertainties (1.98+0.36=2.34 vs. 3.03-0.35=2.68), but their average is approximately 2.5.*

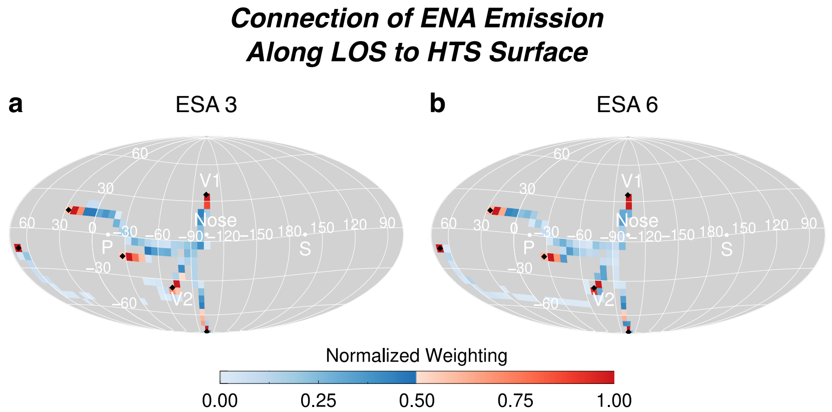

Supplementary Figure 6. *Examples of IBEX lines of sight (LOS) and where they map back to the HTS depending on the HS plasma flow streamlines. Examples are shown for ENAs at energies for ESA 3 (a) and ESA 6 (b) are shown. The colored pixels are positions on the HTS that are connected to the IBEX LOS (black diamond symbols) via bulk plasma flow streamlines in the global MHD simulation. Note that the weights for ESA 3 vs. 6 are slightly different due to the energy-dependent source regions of the ENAs (as one can see, most of the weight originates from similar directions in the sky between ESA 3 and 6, i.e., the red-ish pixels). Because of this, several pixels with weights close to zero appear different in panels a and b, but the flow streamlines themselves are the same.*

Mapping Positions on the HTS to each IBEX Pixel

ENAs created along a particular IBEX line of sight do not originate from a single point on the HTS, but rather from multiple points depending on the bulk flow pattern in the HS[97]. In general, most ENAs integrated over a line of sight originate from PUIs that crossed the HTS within approximately 10°-30° of the line of sight, but this also depends on the region of the sky and deflection of the HS flow downstream of the HTS. Therefore, in the derivation of the best-fit

compression ratio for a particular IBEX line of sight (or pixel) in the sky (see "Finding the Best-fit Compression Ratio at the HTS" in Methods), we minimize over the normalized sum of multiple HTS foot points that contribute to the pixel. We utilize HS plasma flow streamlines from our global MHD simulation to mimic this effect. An example sky map showing several pixels in the sky and where they connect to the HTS is shown in Supplementary Figure 6. The weighting given to each HTS position (colored pixels) is based on the contribution it makes to the total ENA flux from the IBEX pixel (black diamond symbols), i.e., the ENA flux produced from line of sight (LOS) element dl by the local HS proton distribution which propagated from a point on the HTS surface. The reversal of this, i.e., the contribution of one HTS foot point to multiple IBEX pixels, is used in our global minimization scheme.

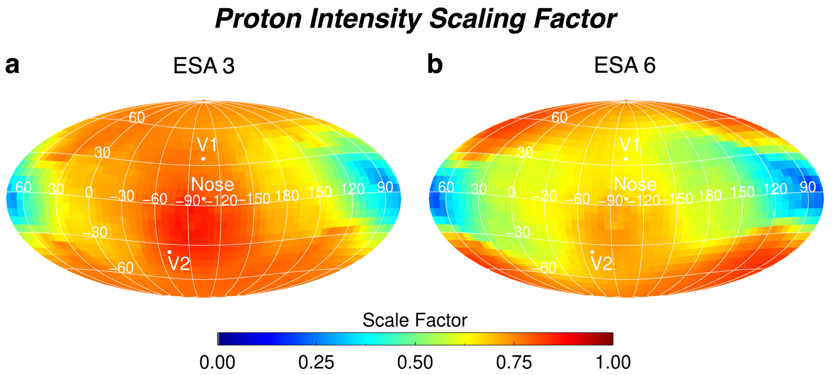

Supplementary Figure 7. *Example sky maps of the intensity scaling factors that we scale the IBEX proton fluxes with as a function of ESA passband, in order to "undo" the evolution of the proton distribution through the HS.*

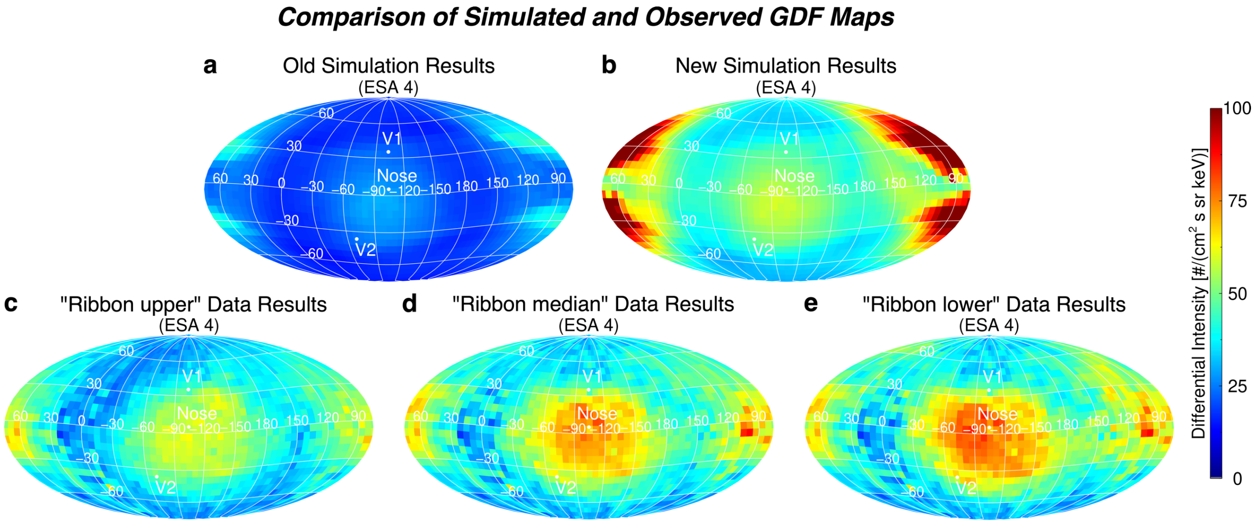

Supplementary Figure 8. *Examples of simulated and IBEX observation-based maps of the GDF in 2009-2011 for ESA 4 (~1.7 keV). The ENA simulation results are shown from two methods: first from the methodology in Zirnstein et al.*[1] *(panel a) and from the methods described in this study (panel b). The observation-based maps are shown in panels c-e. Note that the inclusion of the velocity diffusion effect increased the flux globally such that the simulation results compare much better to the data than in some previous studies*[98-100]*. The oversaturation of simulated fluxes from the mid-latitude, north/south tailward directions is not surprising, as they are due to the fast SW propagating down the heliotail in our steady-state MHD simulation, without the cyclic transitions from fast to slow to fast, etc.,*

SW. The observational data are from IBEX Data Release 18 as validated in McComas et al.[101], based on the ribbon separation methods by Beesley et al.[102]

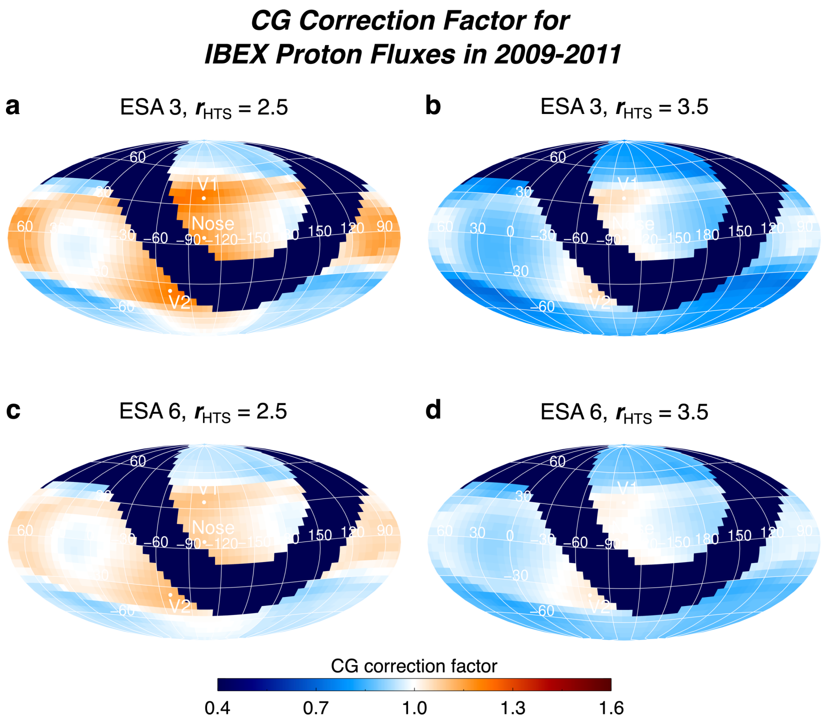

Supplementary Figure 9. *Examples of the CG correction factor as a function of ESA and compression ratio for 2009-2011.*

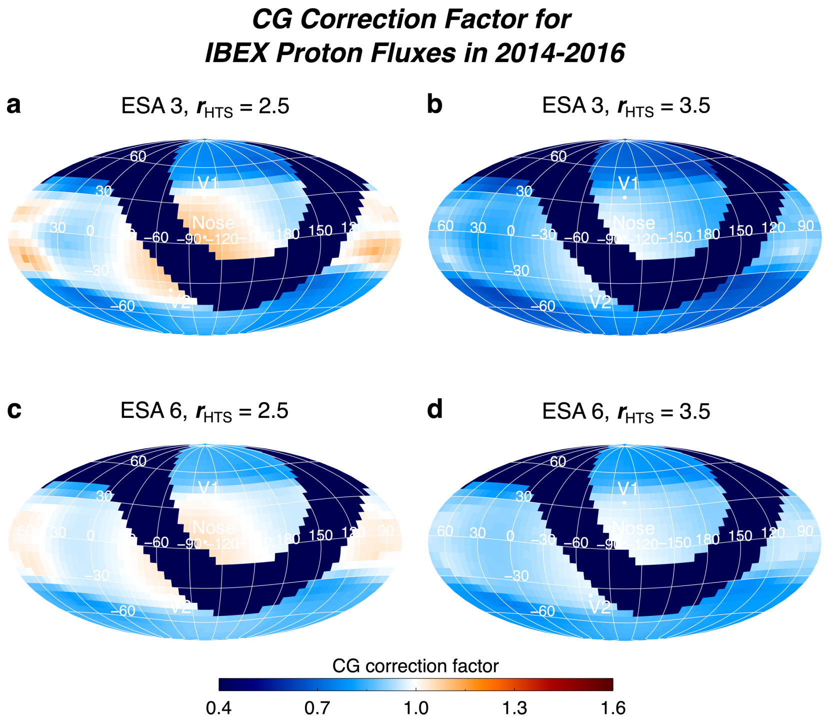

Supplementary Figure 10. *Similar to Figure 9, except for 2014-2016.*

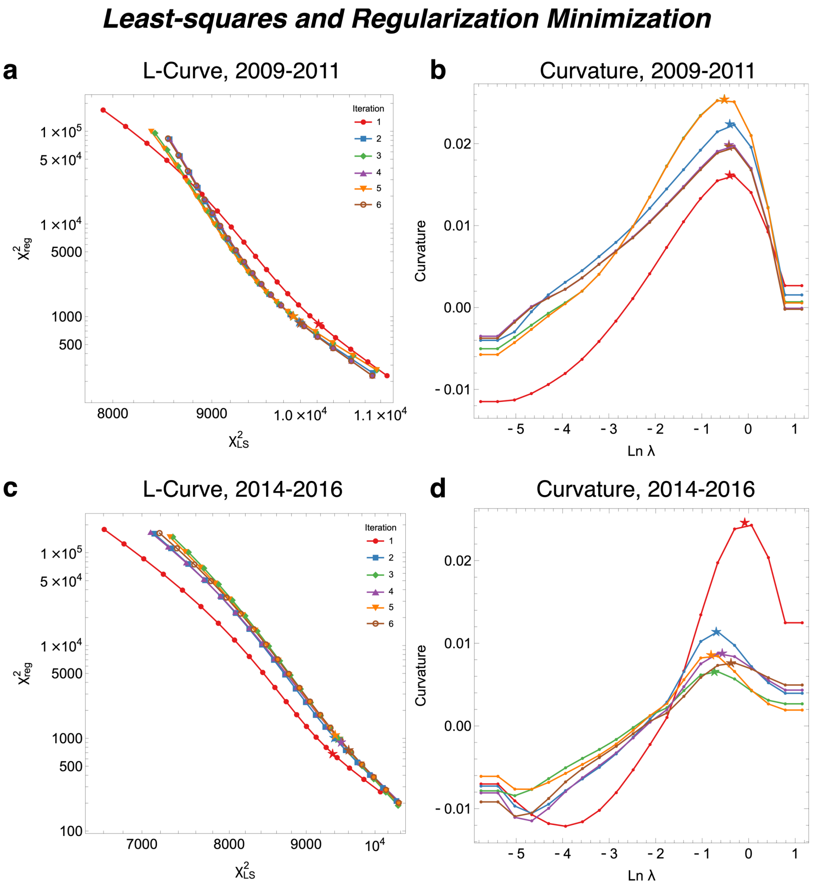

Supplementary Figure 11. *Example of the L-curve (a,c) and its curvature (b,d) used to determine the best-fit compression ratio simultaneously for all pixels in a map. These results are from the nominal case (L1), shown for time periods 2009-2011 (a,b) and 2014-2016 (c,d). The point of maximum curvature is shown as the star in both panels. Note that the stars in panels b and d do not necessarily lie on the curve because the point of maximum curvature is found by fitting a Gaussian function to the 5 points nearest to the peak.*

References

87. Zirnstein, E. J., Dayeh, M. A., Heerikhuisen, J., McComas, D. J. & Swaczyna, P. Heliosheath Proton Distribution in the Plasma Reference Frame. *Astrophys. J. Suppl. Ser.* **252**, 26 (2021).

88. Stone, E. C. *et al.* Voyager 1 Explores the Termination Shock Region and the Heliosheath Beyond. *Science* **309**, 2017–2020 (2005).

89. Stone, E. C. *et al.* An asymmetric solar wind termination shock. *Nature* **454**, 71–74 (2008).

90. Porowski, C., Bzowski, M. & Tokumaru, M. A New 3D Solar Wind Speed and Density Model Based on Interplanetary Scintillation. *Astrophys. J. Suppl. Ser.* **259**, 2 (2022).

91. Zirnstein, E. J. *et al.* Oblique and rippled heliosphere structures from the Interstellar Boundary Explorer. *Nat. Astron.* **6**, 1398–1413 (2022).

92. Swaczyna, P. *et al.* Density of Neutral Hydrogen in the Sun's Interstellar Neighborhood. *Astrophys. J.* **903**, 48 (2020).

93. Zirnstein, E. J. *et al.* Local Interstellar Magnetic Field Determined from the Interstellar Boundary Explorer Ribbon. *Astrophys. J. Lett.* **818**, L18 (2016).

94. Funsten, H. O. *et al.* The Interstellar Boundary Explorer High Energy (IBEX-Hi) Neutral Atom Imager. *Space Sci. Rev.* **146**, 75–103 (2009).

95. Dialynas, K. *et al.* Combined ∼10 eV to ∼344 MeV Particle Spectra and Pressures in the Heliosheath along the Voyager 2 Trajectory. *Astrophys. J. Lett.* **905**, L24 (2020).

96. Decker, R. B. *et al.* Mediation of the solar wind termination shock by non-thermal ions. *Nature* **454**, 67 (2008).

97. Shrestha, B. L., Zirnstein, E. J., Heerikhuisen, J. & Zank, G. P. Strength of the Termination Shock Inferred from the Globally Distributed Energetic Neutral Atom Flux from IBEX. *Astrophys. J. Suppl. Ser.* **254**, 32 (2021).

98. Zirnstein, E. J. *et al.* Structure of the Heliotail from Interstellar Boundary Explorer Observations: Implications for the 11-year Solar Cycle and Pickup Ions in the Heliosheath. *Astrophys. J.* **836**, 238 (2017).

99. Kornbleuth, M., Opher, M., Michael, A. T. & Drake, J. F. Globally Distributed Energetic Neutral Atom Maps for the "Croissant" Heliosphere. *Astrophys. J.* **865**, 84 (2018).

100. Baliukin, I. I., Izmodenov, V. V. & Alexashov, D. B. Heliospheric energetic neutral atoms: Non-stationary modelling and comparison with IBEX-Hi data. *Mon. Not. Royal Astron. Soc.* **499**, 441–454 (2020).

101. McComas, D. J. *et al.* Fourteen Years of Energetic Neutral Atom Observations from IBEX. *Astrophys. J. Suppl. Ser.* **270**, 17 (2024).

102. Beesley, L. J. *et al.* Statistical methods for partitioning ribbon and globally-distributed flux using data from the Interstellar Boundary Explorer. Preprint at https://doi.org/10.48550/arXiv.2302.03089 (2023).
